# Microbiota–Gut–Brain Axis Interactions with CNS Drugs: A Critical Mechanistic Review of Biopharmaceutical, Pharmacokinetic, and Pharmacodynamic Evidence

**DOI:** 10.3390/pharmaceutics18101276

**Published:** 2026-10-09

**Authors:** David Stepensky

**Affiliations:** Department of Clinical Biochemistry and Pharmacology, Faculty of Health Sciences, Ben-Gurion University of the Negev, Beer-Sheva 84105, Israel; davidst@bgu.ac.il; Tel.: +972-8-6477381

**Keywords:** microbiota–gut–brain axis, CNS drugs, microbiome, biopharmaceutics, pharmacokinetics, pharmacodynamics, levodopa, sertraline, amitriptyline, olanzapine, evidence grading

## Abstract

**Background/Objectives**: The microbiota–gut–brain axis (MGBA) modifies central nervous system (CNS) pharmacotherapy by influencing the biopharmaceutics, pharmacokinetics (PK), and pharmacodynamics (PD) of drugs, while CNS drugs reciprocally reshape the MGBA. This review examines these interactions and their clinical relevance, explicitly grading the strength of the evidence. **Methods**: This narrative, mechanistically oriented review focuses on four oral drugs representing distinct mechanistic archetypes: levodopa/carbidopa (biopharmaceutical-dominant), olanzapine (PK-dominant), sertraline (PD-dominant), and amitriptyline (mixed). PubMed/MEDLINE, Google Scholar, and Google were searched up to 1 September 2026; of approximately 260 records screened, 64 were cited. No PRISMA reporting or dual screening was performed. Findings were assigned to five evidence tiers (L1–L5) and classified as associative or causal. **Results**: For levodopa/carbidopa, fecal tyrosine decarboxylase (*tdc*) gene abundance correlates with daily dose requirement (human observational), and *Helicobacter pylori* eradication or treatment of small intestinal bacterial overgrowth improves levodopa exposure or motor outcomes (human interventional). For olanzapine, microbiota depletion increased bioavailability ~1.8-fold in rats (single study), and microbiome-dependent weight gain is causal in rodents, whereas human data are phenomenological only. For sertraline, antimicrobial activity and fecal depletion are demonstrated in vitro; the pharmacodynamic blockade derives from fluoxetine models. For amitriptyline, antimicrobial and anticholinergic effects are preclinical, and the ATLANTIS trial demonstrates efficacy in irritable bowel syndrome without measuring the microbiome. **Conclusions**: The microbiome is a measurable modifier of CNS drug fate, but few interactions are clinically actionable. Progress requires prospective trials with standardized MGBA biomarkers, microbiome-informed PK/PD models, and independent replication.

## 1. Introduction

### 1.1. Bidirectional Communication Within the Microbiota–Gut–Brain Axis

The Microbiota–Gut–Brain Axis (MGBA) is a complex, bidirectional communication network (see Figure 1) that integrates the enteric nervous system and the gastrointestinal (GI) tract with the central nervous system (CNS) [1,2]. Throughout this review, *microbiota* refers to the community of living microorganisms residing in the gut, *microbiome* refers collectively to these organisms together with their genes and metabolic products, and *MGBA* denotes the entire bidirectional signaling system connecting this microbial ecosystem to the CNS; these terms are used consistently in this sense throughout the manuscript. This axis coordinates physiological and behavioral processes through four primary signaling pathways: neural (primarily via the vagus nerve), endocrine (the hypothalamic–pituitary–adrenal [HPA] axis and gut hormones), immune (cytokines and microglial activation), and metabolic/humoral (short-chain fatty acids [SCFAs], tryptophan metabolites, and microbial neuroactive compounds) [2,3]. Bidirectionality is the defining feature of this system: while microbial signals and metabolites modulate CNS function and maturation, CNS-driven changes in gut motility, secretion, and permeability simultaneously reshape the microbial ecosystem [1,2,4]. Consequently, the axis acts as a single integrated regulatory system in which a perturbation in one organ, through disease, stress, or pharmacological treatment, is capable of shifting the system to a new steady state [2,3].

### 1.2. The Role of MGBA as a Regulator of CNS Drug Exposure and Response

Beyond its role in disease pathogenesis, the MGBA has emerged as an important regulator of CNS pharmacotherapy [5,6] (see Figure 1). The intestinal microbiome and its metabolites are important modulators of drug biofate, in an area increasingly known as pharmacomicrobiomics [5,6]. In the biopharmaceutical domain, the microbiome acts as a pre-systemic filter, in which bacterial enzymes can chemically transform drugs (metabolism) or sequester them intracellularly (bioaccumulation) before they reach the systemic circulation [5,7]. Pharmacokinetically, microbial signals regulate host ADME (absorption, distribution, metabolism, and excretion) by modulating the integrity of the GI epithelium, the activity of hepatic and intestinal cytochrome P450 (CYP450) enzymes, the integrity of the blood–brain barrier (BBB), and the activity of efflux transporters such as P-glycoprotein, etc. [5,6,8]. Furthermore, the MGBA affects pharmacodynamic processes via gut-driven neuroinflammation, altered neurotransmitter precursor flux, and changes in the expression and activity of receptors and other drug targets, leading to alterations in the efficacy and safety of drug treatment [5,9].

### 1.3. CNS Drugs as a Case Study for Host–Microbiome Interactions

CNS drugs offer a unique and high-value model for studying host–microbiome–drug interactions because their disposition and activity are shaped by multiple gut-related processes [5,10]. Most centrally acting agents target receptor systems (e.g., serotonergic and dopaminergic) that are simultaneously expressed in the CNS and the enteric nervous system, and that affect the MGBA at several locations [11,12]. Furthermore, many psychotropic medications, such as selective serotonin reuptake inhibitors (SSRIs) and tricyclic antidepressants (TCAs), possess intrinsic antimicrobial activity, suppressing numerous human gut bacterial strains [13,14].

In the foundational high-throughput screen, which tested >1000 marketed compounds against 40 representative commensal gut strains, 24% of the 835 human-targeted drugs across all therapeutic classes inhibited the growth of at least one strain in vitro, with chemically diverse antipsychotics overrepresented among the active compounds [14]. A more recent, class-restricted follow-up from the same laboratory evaluated 1614 centrally acting agents (antidepressants, antipsychotics, and other psychotropics) against an expanded panel of gut commensals, pathobionts, and pathogens; approximately 14% of these compounds exhibited antibacterial activity against at least one strain, with butyrate- and propionate-producing taxa disproportionately affected. These updated, class-focused estimates constitute in vitro/mechanistic (Level 4) evidence and should not be extrapolated as validated clinical effect sizes. Importantly, these population-level inhibition estimates are lower than the strain suppression fractions reported for individual agents such as sertraline and amitriptyline in dedicated single-drug assays (see Section 6.3 and Section 6.4). Thus, the 14–24% figures describe the proportion of drugs active against at least one organism in a broad bacterial panel, whereas the ~73% figures for sertraline and amitriptyline describe the proportion of strains suppressed by those two specific drugs; these are distinct metrics and are not directly comparable.

Thus, drugs modify the gut microbiome, potentially contributing to treatment resistance or metabolic side effects, such as the weight gain observed with antipsychotics [13,15]. Therefore, microbiome and MGBA-related factors should be considered, alongside classical pharmacokinetic and pharmacodynamic considerations, to describe the biofate of CNS drugs appropriately [16].

### 1.4. Distinguishing Clinically Important Factors from Preclinical Data

A primary objective of this critical mechanistic review is to distinguish established human clinical evidence from preclinical findings and mechanistic considerations [17,18]. Animal models, such as germ-free and gnotobiotic rodents, have been indispensable for research on MGBA, but species-specific differences in microbial composition and drug metabolism can lead to improper extrapolation of these data to clinical settings [17,18]. This review emphasizes the potential therapeutic relevance of experimental data, with particular attention to the magnitude, reproducibility, and clinical significance of specific findings, and assigns each major mechanism an explicit evidence tier designation (see below). We aimed to analyze model CNS drugs and their interactions with the microbiome and other MGBA components, to define the clinical importance of these interactions and to describe their potential role in precision neuropharmacology [19,20,21]. This knowledge is essential for the future validation of standardized MGBA biomarkers and for the eventual implementation of MGBA-informed clinical guidelines for optimized individualized treatment.

### 1.5. Rationale for Drug Selection and the Four Mechanistic Archetypes

This review focuses on four orally administered CNS drugs: levodopa/carbidopa, olanzapine, sertraline, and amitriptyline. Each of the four drugs was chosen because it represents a distinct, well-characterized mechanistic archetype through which the MGBA can dominate a drug’s biofate and response, allowing this review to illustrate the full breadth of MGBA–drug interactions using a tractable number of agents rather than providing an exhaustive but shallower catalog of every CNS drug with reported microbiome interactions. The four archetypes, elaborated mechanistically in Section 6 and summarized in the Discussion (Section 7.1), are as follows: (i) levodopa/carbidopa illustrates a biopharmaceutical-dominant archetype, in which pre-systemic microbial metabolism in the gut lumen is the principal and best-characterized determinant of clinically relevant variability in drug exposure; (ii) olanzapine illustrates a pharmacokinetic-dominant archetype, in which microbiome–host crosstalk regulates intestinal and hepatic drug-metabolizing enzymes and thereby systemic bioavailability, while the drug reciprocally drives microbiome-mediated metabolic toxicity; (iii) sertraline illustrates a pharmacodynamic-dominant archetype, in which the clinically decisive MGBA effect operates largely downstream of systemic exposure, through microbiome-dependent modulation of neuroinflammation and the kynurenine pathway; and (iv) amitriptyline illustrates a mixed mechanism archetype in which biopharmaceutical (motility-dependent luminal exposure), pharmacokinetic, and pharmacodynamic (direct CNS and enteric neuromodulation) factors act in combination.

Other candidate drugs with comparable or, in some domains, more extensive MGBA literature (e.g., clozapine, fluoxetine, lithium, valproate, or benzodiazepines) were considered but not selected as primary case studies. Clozapine and lithium/valproate have informative but comparatively fragmented human PK data. Fluoxetine has rich preclinical MGBA data, but as an SSRI, its mechanistic profile substantially overlaps with that of sertraline; accordingly, fluoxetine is cited within the sertraline sections as supportive, class-level preclinical evidence rather than as a standalone case study.

## 2. Framework and Terminology

### 2.1. Study Design and Literature Search Strategy

This is a narrative, mechanistically oriented review of MGBA–drug interactions, focusing on four orally administered CNS drugs: levodopa/carbidopa, olanzapine, sertraline, and amitriptyline. PubMed/MEDLINE and Google Scholar were searched, supplemented by general Google searches. Boolean combinations of the following terms were used: (“microbiome” OR “microbiota” OR “gut–brain axis” OR “microbiota–gut–brain axis”) AND (“CNS drug” OR “psychotropic” OR “levodopa” OR “carbidopa” OR “olanzapine” OR “sertraline” OR “amitriptyline”) AND (“pharmacokinetic” OR “pharmacodynamic” OR “bioavailability” OR “drug metabolism”). No lower date limit was applied; English-language full-text or abstract records were eligible. The final search date was 1 September 2026. Reference lists of key reviews were hand-searched for additional primary sources. The Boolean strings above were applied to PubMed/MEDLINE and Google Scholar, which served as the principal, reproducible sources; general Google searches were used only as a supplementary means of identifying additional records (e.g., recent publications), and all cited records were checked against their original publications. The number of cited records (64) reflects the deliberately selective, archetype-based scope of this review (four drugs), and the preference for citing primary studies and key reviews rather than redundant replications; the review aims at mechanistic synthesis and is not exhaustive.

Eligible studies included experimental in vitro and ex vivo investigations, animal models, and human observational or interventional studies that reported mechanistic data on microbiome–drug interactions and, where available, quantitative effect sizes (e.g., fold changes in drug exposure, biomarker levels, or clinical scores).

Literature identification, screening, full-text review, data extraction, and synthesis were performed by a single author (D.S.). Approximately 260 unique records were identified and screened by title/abstract; approximately 120 were retrieved as full text for mechanistic relevance; and 64 were ultimately selected for citation based on mechanistic informativeness, methodological quality, and recency, with redundant preclinical replications of already-cited mechanisms generally not cited separately.

Selection of the 64 cited records was based on the relevance of their topic and content to MGBA–drug interactions, with emphasis on review articles and on primary studies involving the four selected prototype drugs, rather than on the direction of their findings; records reporting negative or null microbiome–drug associations were not excluded on that basis and are cited where identified (e.g., the absence of independent replication for the olanzapine bioavailability finding, Section 4.2, and the lack of a demonstrated sertraline-specific pharmacodynamic blockade, Section 6.3.2). Manuscripts addressing non-CNS drugs were excluded, as were most manuscripts addressing CNS drugs other than the four selected prototypes, with the exception of a limited number of references retained for broader mechanistic or comparative context (e.g., fluoxetine as a class-level SSRI comparator, Section 1.5). Because the underlying primary literature in this field is itself disproportionately composed of positive, mechanistically confirmatory findings, negative or null studies are almost certainly underrepresented among the records available for citation irrespective of screening approach; this source of publication bias, and its implications for the conclusions drawn here, is discussed explicitly in Section 7.4.

Given the heterogeneity of study designs and endpoints, no formal meta-analysis or quantitative pooling was performed. Instead, quantitative data were synthesized narratively, with emphasis on: (a) the mechanism and magnitude of MGBA–drug interactions, (b) the consistency of findings across independent studies and models, and (c) the translational relevance of preclinical data to human CNS pharmacotherapy. The results of the literature search and mechanistic analyses are synthesized in Supplementary Tables S1–S4, while the principal findings are presented and discussed in the subsequent sections of the manuscript.

### 2.2. Establishing a Clear Conceptual Framework: MGBA-on-Drug vs. Drug-on-MGBA Effects

To accurately describe the complex interactions within the MGBA, this review establishes a conceptual framework based on the bidirectionality of host–microbiome–drug relationships [22]. We distinguish between two primary vectors of interaction:

MGBA-on-Drug Effects: this vector encompasses the mechanisms by which the intestinal microbiota, its metabolites, and other MGBA components affect pre-systemic biotransformation and sequestration of a drug by the gut lumen bacteria [7,10], alter the pharmacokinetics (PK) of a drug through changes in gut permeability, gut wall and hepatic metabolism, and permeability to the brain and to other organs, with the resulting changes in drug absorption, distribution, metabolism, and/or excretion (ADME), and modify pharmacodynamic (PD) processes.

Drug-on-MGBA Effects: orally administered medications frequently exhibit intrinsic antimicrobial activity. In the foundational high-throughput screen of >1000 marketed compounds, approximately 24% of the 835 human-targeted drugs tested inhibited the growth of at least one commensal bacterial strain in vitro [14]. A subsequent, psychotropic-focused follow-up screen refined this estimate to approximately 14% within a class-restricted panel of centrally acting agents (see Section 1.3) [23]. By directly suppressing susceptible taxa, these drugs can reshape gut microbiome composition and perturb microbiota-dependent metabolic pathways, with downstream consequences across multiple MGBA branches. These microbiome-mediated effects may also be accompanied by drug-induced alterations in the enteric nervous system, gastrointestinal motility, barrier integrity, and other aspects of GI function. Together, these reciprocal drug–GBA interactions (see Supplementary Table S1) create dynamic feedback loops in which the drug remodels the very ecosystem that governs its subsequent metabolism, efficacy, and toxicity [24,25].

### 2.3. Categorizing Interactions into Biopharmaceutical, Pharmacokinetic, and Pharmacodynamic Domains

This manuscript categorizes MGBA–drug interactions into three distinct domains to facilitate mechanistic description and understanding of drug–MGBA interactions (see Supplementary Table S1):

Biopharmaceutical Domain: Focuses on mutual interactions occurring in the intestinal lumen before systemic absorption, including microbial metabolism (e.g., the decarboxylation of levodopa), microbial bioaccumulation (e.g., the sequestration of sertraline), changes in the composition and pH of GI fluids that alter the solubility of pH-dependent CNS agents, and changes in GI motility that affect the GI transit of CNS drugs, etc. [24,25].

Pharmacokinetic Domain: Examines the modulation of host ADME processes by microbial signals, including changes in the integrity of the GI epithelium, regulation of hepatic and intestinal CYP450 enzymes (e.g., CYP3A4, CYP1A2), interference with first-pass metabolism, and the regulation of BBB integrity by metabolites such as SCFAs [5,16,19].

Pharmacodynamic Domain: Addresses how the microbiome dictates the biological context in which a drug acts, in relation to neuroimmune signaling, microglial activation, and the modulation of neurotransmitter precursor flux (e.g., the kynurenine pathway), which can functionally blunt or attenuate the behavioral response to therapies like SSRIs [9,13].

### 2.4. Evidence Grading and Assessment of the Magnitude and Clinical Significance of the Drug–MGBA Interactions

Classification with an explicit five-tier ordinal evidence-grading scale was applied consistently to individual mechanisms and quantitative findings throughout the text (Section 3, Section 4, Section 5 and Section 6) and in Supplementary Tables S1–S4:

Level 1—Human randomized or other controlled interventional trial.
Level 2—Human observational, cohort, cross-sectional, or single-arm/open-label interventional study.
Level 3—Animal (in vivo) model, including germ-free, gnotobiotic, antibiotic depletion, and FMT rodent studies.
Level 4—In vitro/ex vivo/mechanistic study, including bacterial culture, fecal suspension, and enzymatic assays using human- or animal-derived material outside a living host.
Level 5—Hypothetical or not yet experimentally tested mechanism, proposed on the basis of pathway plausibility or indirect analogy.

For each major interaction discussed, we explicitly address, where data permit: (a) the magnitude of the effect; (b) its reproducibility across independent studies or models; (c) its relevance to clinical dosing decisions; and (d) whether therapeutic drug monitoring or microbiome-targeted intervention is currently clinically feasible.

By applying this graded framework to distinguish established human clinical evidence from preclinical findings, we aim to delineate which drug–MGBA interactions are presently clinically actionable and which require further validation through dedicated clinical trials combining microbiome profiling with quantitative pharmacokinetic and pharmacodynamic endpoints [17,18].

### 2.5. Causality Versus Association: An Explicit Framework

A central challenge in MGBA pharmacology is discriminating between microbiome alterations that causally drive altered drug responses and those that are merely associated with, or secondary to, the drug response or the underlying disease state. To address this, we applied three interpretive criteria to the evidence synthesized in Section 3, Section 4, Section 5 and Section 6 and tabulated in Supplementary Tables S2 and S3:

Temporal precedence: studies that quantify microbiome composition or specific bacterial gene abundances prior to drug administration or symptom onset, and demonstrate that these baseline measures predict subsequent drug exposure or clinical response, provide stronger evidence for an upstream, causal role of the microbiome than cross-sectional designs in which microbiome and outcome are assessed concurrently.

Directionality of the perturbation: experimental designs that directly manipulate the microbiome, such as germ-free colonization, antibiotic-mediated depletion, fecal microbiota transplantation (FMT), or selective bacterial enzyme inhibition (e.g., AFMT), and subsequently document altered drug exposure or a pharmacodynamic effect constitute direct evidence of causality. In contrast, associations based solely on correlations between baseline microbiome features and clinical outcomes are compatible with multiple causal architectures: the microbiome shift may be a cause, a consequence, or a shared downstream effect of a third variable (e.g., dietary improvement, increased physical activity, or reduced systemic inflammation accompanying clinical recovery). Such findings are therefore interpreted as associative unless supported by interventional or mechanistic data.

Mechanistic validation: causal inference is strongest when the proposed microbiome → drug pathway has been experimentally resolved at the molecular level. Conversely, reports describing only a correlation between a microbial taxon and a pharmacokinetic or clinical endpoint, in the absence of interventional or enzymatic evidence, are classified as associative and explicitly flagged in the text using cautious language (e.g., “correlates with” or “is associated with” rather than “causes” or “reduces”).

## 3. Biopharmaceutical Mechanisms: The Pre-Systemic Factors in the Gut Lumen

### 3.1. Microbial Metabolism: Chemical Transformation in the Gut Lumen

Gut microorganisms possess a diverse enzymatic repertoire that can chemically transform CNS drugs, often leading to their inactivation or to the production of pharmacologically active metabolites [5,26]. This mechanism is prominent for levodopa, a prodrug used in the management of Parkinson’s disease, which is susceptible to premature decarboxylation into dopamine by bacterial tyrosine decarboxylases (TDC), primarily from species such as *Enterococcus faecalis*—a mechanism demonstrated in vitro and corroborated by human observational data (Level 4/Level 2) [17,21]. Carbidopa, which is commonly co-administered with levodopa to prevent its metabolism to dopamine outside the CNS, is inefficient in the GI lumen because it is 14,000–19,000-fold less potent against microbial TDC than against human aromatic L-amino acid decarboxylase (Level 4, enzymatic assay) [21,27]. Consequently, standard doses of carbidopa fail to prevent microbial levodopa loss in patients with high *tdc* gene abundance (Level 2) [18]. This luminal metabolism diminishes the effectiveness of levodopa treatment because dopamine does not efficiently penetrate the CNS even when it reaches the systemic circulation from the GI lumen [20,28]. Subsequent metabolism by *Eggerthella lenta* can transform the luminal dopamine into m-tyramine, a metabolite associated with adverse hypertensive effects (Level 4/Level 2, genotype correlative) [20,29]. Other pathways of luminal levodopa metabolism include the deamination of levodopa by *Clostridium sporogenes* into 3-(3,4-dihydroxyphenyl)propionic acid, which has been shown to inhibit local intestinal motility in ex vivo/animal systems (Level 3/4) [27].

### 3.2. Bioaccumulation of Drugs by Gut Bacteria

Beyond chemical transformation, bacteria can influence drug exposure through bioaccumulation, in which drug molecules are sequestered intracellularly without being chemically modified (Level 4, in vitro) [7]. In principle, this sequestration may lower the fraction of drug available for absorption [30]. This mechanism is prominent for sertraline (see Section 6.3.1) and for duloxetine, an antidepressant drug, that undergoes bioaccumulation by several commensal species, including *Streptococcus salivarius* and *Bacteroides uniformis* [7,30], with possible downstream effects on other bacterial taxa in the gut lumen that interact with these species [7,31]. As a result, the bioavailability of these drugs may be affected by the individual patient’s microbiome and its changes, potentially contributing to the interindividual variability observed in their antidepressant efficacy. However, the clinical (human in vivo) magnitude of this effect has not been established and remains a Level 4/Level 5 (mechanistic/hypothetical) extrapolation [7,30].

### 3.3. Intestinal Lumen Contents: Changes in pH and Solubility

The contents of the GI lumen, particularly the volume and composition of fluid and its pH, affect the dissolution and absorption of CNS drugs, many of which are weak acids or weak bases [5,6]. Microbial fermentation of dietary fibers produces SCFAs and organic acids that can significantly lower luminal pH [13,32]. These shifts in acidity alter the ionization state and solubility of pH-sensitive agents, affecting drug absorption and systemic exposure in states of dysbiosis (Level 3/4, mechanistic) [5]. Furthermore, the use of proton pump inhibitors (PPIs), H_2_ blockers, or antacids increases gastric pH, which not only affects drug dissolution but also promotes the overgrowth of *Enterococcus* species, thereby intensifying the microbial degradation of drugs like levodopa (Level 2, human observational) [28,33].

### 3.4. Gastrointestinal Motility

Gastrointestinal motility determines the residence time that a drug spends in specific gut segments, dictating the duration of exposure to microbial enzymes [5]. This interaction is bidirectional: CNS drugs may alter motility, which in turn reshapes the microbiome [6,22]. Amitriptyline and other tricyclic antidepressants possess potent anticholinergic properties that slow GI transit (Level 3, animal/experimental) [34,35]. This slowed motility can create a self-reinforcing loop in which increased residence time allows for greater microbial sequestration or metabolism, potentially reducing systemic exposure—a mechanistic hypothesis (Level 5) that has not been directly tested in a dedicated PK study in humans [22].

Gut-derived serotonin, originating from the enteric nervous system, serves as a primary regulator of peristalsis [3,11]. Orally administered SSRIs increase serotonin levels in the gut, leading to side effects such as diarrhea, which may reduce the time available for drug absorption (Level 2, clinical observation) [11,36]. Pathological states such as Parkinson’s disease-associated constipation further complicate this process by delaying drug delivery to the primary absorption sites in the jejunum, thereby contributing to delayed-on motor fluctuations (Level 2) [29,33].

## 4. Pharmacokinetic Mechanisms: ADME Modulation

### 4.1. Altered Gut Epithelial Permeability (“Leaky Gut”)

The integrity of the intestinal epithelial barrier dictates the efficiency of both passive and active drug transport [9,37]. In a healthy state, tight junction proteins (e.g., occludin, claudin-1, zonula occludens-1) regulate paracellular flux, a process supported by microbially derived butyrate (Level 3/4) [32,38,39]. Pathological perturbations such as chronic stress, systemic inflammation, or diet-related factors can induce “leaky gut,” characterized by the opening of these junctions (Level 3) [9,13,40]. Increased gut epithelial permeability leads to the absorption of bacterial products like lipopolysaccharide (LPS) into the circulation, triggering neuroinflammatory cascades that can attenuate drug response (Level 3/4) [13].

The microbiome also regulates the expression of host efflux transporters, such as P-glycoprotein (P-gp), in the gut epithelium. Intestinal P-gp is downregulated in the absence of a healthy microbiome (up to four-fold in germ-free models; Level 3) [16] or by bacteria, such as *Bacteroides thetaiotaomicron*. Other bacteria, such as *Eggerthella lenta*, produce soluble P-gp inhibitors [31]. Each of these effects can enhance the bioavailability of orally administered P-gp substrates (Level 3/4, animal/in vitro, not yet confirmed in human PK studies) [16,31].

### 4.2. First-Pass and Systemic Metabolism in the Gut Wall and Liver

Microbiome-derived signals regulate the activity of host drug-metabolizing enzymes, particularly the cytochrome P450 (CYP450) and uridine diphosphate-glucuronosyltransferase (UGT) superfamilies (Level 3/4) [17,19]. Microbial metabolites, such as p-cresol, can compete for host metabolic enzymes, while other signals regulate the expression of hepatic CYP3A4 and CYP1A2 via nuclear receptors such as farnesoid and pregnane X receptors [16,17]. Microbiota depletion in rats via broad-spectrum antibiotics precipitates a 1.8-fold increase in the systemic bioavailability of olanzapine (Level 3, single animal study) [19] attributed primarily to downregulation of duodenal UGT1A3 (the rat functional equivalent of human UGT1A4), the principal enzyme responsible for olanzapine glucuronidation [19]. This finding has not yet been replicated in an independent animal cohort or examined in a human PK study and should be regarded as hypothesis-generating (Level 5 for human translation) rather than an established clinical PK interaction.

Dysbiosis-induced systemic inflammation provides an additional, indirect mechanism by which the MGBA affects drug metabolism. Elevated systemic levels of pro-inflammatory cytokines (e.g., IL-6, TNF-α) suppress hepatic metabolic activity, mostly via CYP450 rather than UGT enzymes, potentially leading to enhanced accumulation and toxicity risk of drugs eliminated preferentially by CYP450 enzymes (Level 5, mechanistic extrapolation) [22].

### 4.3. Enterohepatic Cycling and Microbial Deconjugation

The gut microbiome facilitates the reactivation of some drugs that undergo enterohepatic cycling, effectively extending their systemic half-life (Level 3/4) [31,41]. Some antipsychotics and antidepressants undergo hepatic phase II glucuronidation to form glucuronide conjugates that accumulate in the bile and are excreted into the intestinal lumen [16,19]. Gut bacterial glucuronidases deconjugate these metabolites, regenerating the active parent drug in the gut lumen, which undergoes reabsorption [16,41]. Changes in the gut microbiome, e.g., in dysbiosis or induced by antibiotics, alter the recycling efficiency of drugs that undergo enterohepatic cycling, contributing to variability in drug exposure and in the balance of desired vs. undesired effects. This remains a largely mechanistic (Level 4/5) inference rather than a quantified human clinical finding.

### 4.4. Blood–Brain Barrier Integrity and CNS Drug Delivery

The microbiome is an important determinant of BBB integrity and permeability (Level 3, animal) [38,42]. Microbially derived SCFAs, particularly butyrate and propionate, upregulate the expression of tight junction proteins such as occludin and claudin-5, contributing to BBB function [3,32,42]. Germ-free mice consistently exhibit increased BBB permeability, a defect that can be reversed through colonization with SCFA-producing bacteria or direct butyrate supplementation (Level 3) [32,38].

Conversely, microbial metabolites such as trimethylamine N-oxide and dysbiosis-triggered systemic inflammation can disrupt these tight junctions, increasing toxin infiltration and drug penetration from plasma to the brain (Level 3/4) [38,42]. As a result, systemic drug concentrations may only partially reflect brain drug concentrations, and gut microbiome alterations may affect brain/plasma drug concentration ratios—an inference with direct implications for the interpretation of therapeutic drug monitoring, which is based on quantitative assessment of systemic rather than CNS drug concentrations, but has not itself been directly measured in a human MGBA-CNS drug study (Level 5).

## 5. Pharmacodynamic Mechanisms: Changes in Drug Target Occupancy and Pharmacological Effects

The previous sections addressed the biopharmaceutical and pharmacokinetic MGBA factors that alter the concentration of a drug reaching the systemic circulation and the brain. In addition to these factors that affect brain exposure to the drugs, microbiome-related signals modulate central target sensitivity, neuroplasticity, and the stress response system, potentially altering drug efficacy even when CNS exposure to the drug remains unchanged [5]. As emphasized in Section 2.5, human evidence in this domain remains largely correlative, and causal interpretation should be made cautiously [17].

### 5.1. Neuroimmune and Inflammatory Signaling: Microglial Activation and BBB Crosstalk

The gut microbiome shapes the activity of the innate immune system, specifically regulating the maturation and functional state of microglia, the resident macrophages of the CNS (Level 3) [3,42]. In dysbiosis, increased intestinal permeability facilitates the translocation of bacterial LPS and other endotoxins into the systemic circulation (Level 3/4) [9,42]. These molecules trigger the release of pro-inflammatory cytokines, such as IL-6 and TNF-α, which can cross the BBB or stimulate brain-resident immune cells via endothelial signaling (Level 3) [5,22].

Chronic exposure to these gut-derived inflammatory signals results in microglial priming, in which these cells adopt a hyper-responsive phenotype (Level 3, preclinical). [3,9,42]. This pro-inflammatory milieu can diminish the therapeutic response to CNS drugs. For example, neuroinflammation can downregulate BDNF expression and alter serotonergic receptor sensitivity, potentially contributing to antidepressant resistance in major depressive disorder (Level 3/4, with human correlative support discussed in Section 6.3) [9].

### 5.2. Neurotransmitter Precursor Availability

The microbiome significantly influences the pool of circulating amino acids available for central neurotransmitter synthesis (Level 3/4) [6]. Tryptophan, the essential precursor for serotonin, represents a critical node of MGBA–drug interaction [13,43]. Under normal conditions, a fraction of tryptophan is converted to serotonin; systemic inflammation, however, induces the enzyme indoleamine 2,3-dioxygenase, which shunts tryptophan toward the kynurenine pathway (Level 3) [9]. This metabolic shift both reduces the substrate available for serotonin synthesis and increases the production of neurotoxic metabolites such as quinolinic acid, which promotes excitotoxicity and oxidative stress (Level 3/4) [9,43]. Such a neurotoxic background can diminish the pharmacological effects of SSRIs and other serotonergically active drugs.

An additional pathway relevant to catecholamine neuromediators involves the conversion of dietary tyrosine by gut microbiota to 4-ethylphenyl sulfate (4-EPS), a metabolite that has been shown in preclinical models to cross the BBB, disrupt oligodendrocyte maturation, and induce anxiety-like behavior (Level 3, single preclinical study; human relevance not established, Level 5) [38,44].

### 5.3. Vagus Nerve Communication: Direct Neural Signaling Pathway

The vagus nerve allows the microbiome to influence the CNS directly and independently of its metabolic effects (via LPS, tyrosine, tryptophan, etc.) [32,42]. Specialized enteroendocrine cells, termed neuropods, establish direct synaptic contact with vagal afferents, using glutamate and serotonin to relay microbial signals from the gut lumen to the brainstem (Level 3/4) [37,45]. These vagal signals project to the nucleus tractus solitarius and subsequently to limbic regions involved in mood and stress regulation (Level 3) [40,46]. Preclinical experiments demonstrate that the anxiolytic and antidepressant effects of certain psychobiotics (e.g., *Lactobacillus rhamnosus*) are abolished following vagotomy, indicating vagal involvement in MGBA-mediated behavioral change (Level 3, causal within the animal model) [47,48]. This mechanism has not been directly tested in humans, and its clinical relevance for CNS drug pharmacodynamics remains inferential (Level 5).

### 5.4. Neuroplasticity and HPA Axis Modulation: BDNF and Cortisol Feedback

The microbiome is a potent regulator of the hypothalamic–pituitary–adrenal (HPA) axis, the body’s primary stress response system (Level 3) [40]. Landmark studies in germ-free (GF) mice have established that the absence of a microbiome leads to exaggerated HPA reactivity and altered stress-related behavior (Level 3, causal within the model) [40,49]. Dysbiosis-induced HPA hyperactivation leads to elevated cortisol (or corticosterone in rodents), which in turn damages the intestinal barrier and reinforces a pro-inflammatory steady state (Level 3) [40,50].

Central neuroplasticity, often measured by BDNF levels, is similarly sensitive to microbial signals (Level 3) [39]. Preclinical models show that short-chain fatty acids (SCFAs), particularly butyrate, act as histone deacetylase inhibitors that can upregulate hippocampal BDNF expression, potentially enhancing psychotropic treatment efficacy (Level 3) [38,51]. Chronic stress or antibiotic-induced dysbiosis, however, can deplete these neuroprotective SCFAs, reducing central plasticity and attenuating drug effects on neuronal growth and survival (Level 3, all preclinical; no direct human confirmation, Level 5 for clinical translation) [38].

## 6. Drug-Specific Mechanistic Analysis

This section summarizes the available data on interactions between the MGBA and the four drugs selected to represent the mechanistic archetypes introduced in Section 1.5, together with the resulting changes in the biopharmaceutics, pharmacokinetics, and pharmacodynamics of these drugs (see Supplementary Table S2).

### 6.1. Levodopa/Carbidopa

#### 6.1.1. Pre-Systemic Levodopa Metabolism in the Gut Lumen

Levodopa/carbidopa—MGBA interactions are extensive and appear to be clinically important (see Supplementary Table S3). Levodopa is a prodrug that is intended to reach the CNS and be converted to dopamine via aromatic L-amino acid decarboxylase for the management of Parkinson’s disease. To inhibit peripheral activation, carbidopa, an inhibitor of aromatic L-amino acid decarboxylase, is co-administered in the same pill. Despite this, up to 56% of an oral levodopa dose is lost to peripheral metabolism in clinical settings, because of microbial tyrosine decarboxylases (*tdc*) from *Enterococcus* and other gut taxa (Level 2, human observational) [20]. This reflects the 14,000–19,000-fold lower potency of carbidopa against microbial tyrosine decarboxylases relative to human aromatic L-amino acid decarboxylase (Level 4, enzymatic) [21]. Because dopamine cannot cross the BBB, this luminal conversion restricts central drug availability [28]. In human clinical cohorts, this mechanism appears to be clinically important, as fecal *tdc* gene abundance shows a strong positive correlation (r = 0.66) with the daily levodopa dose required for symptomatic relief (Level 2, cross-sectional correlation—associative rather than proven causal, per Section 2.5, though supported by the mechanistic chain established at Level 4) [21]. Sex-based microbiota differences may also affect pre-systemic levodopa metabolism, with ~2.1-fold higher peak concentration (C_max_) and ~1.8-fold higher area under the curve (AUC) reported in females than in male patients (Level 2) [33].

The *tdc* dose correlation (r = 0.66) is among the strongest human microbiome–drug response relationships reported in this literature and has been reported by more than one group (Table S3), supporting reasonable reproducibility; fecal *tdc* quantification (qPCR) is technically feasible today, but no validated clinical assay or dosing algorithm yet exists, so this interaction remains clinically informative but not yet clinically actionable.

#### 6.1.2. Secondary Metabolism of Dopamine in the Lumen and Motility Feedback

Beyond initial decarboxylation, the MGBA involves complex secondary transformations that are largely supported by preclinical and ex vivo evidence [17,29]. Following luminal production, *Eggerthella lenta* can further metabolize it into m-tyramine (Level 4) [20]. Mechanistically, the presence of the Arg506 single-nucleotide polymorphism in *E. lenta* predicts a nearly complete conversion of dopamine into m-tyramine, a metabolite associated with hypertensive and autonomic adverse effects in human populations (Level 2, genotype correlative) [20]. Additionally, residual levodopa can be deaminated by *Clostridium sporogenes* into 3-(3,4-dihydroxyphenyl)propionic acid (Level 4) [27], which inhibits ileal motility, as demonstrated in animal and ex vivo studies (Level 3/4), potentially creating a self-reinforcing loop in which slowed drug transit increases microbial degradation, further reducing systemic bioavailability [27]. The clinical importance of this loop in patients with Parkinson’s disease has not yet been established (Level 5 for human relevance).

#### 6.1.3. Clinical Translation and Pathological Modifiers

Human observational data indicate that alterations in the gut microbiome, such as small intestinal bacterial overgrowth (SIBO), reported in 54–67% of patients with Parkinson’s disease, and *Helicobacter pylori* infection, affect levodopa bioavailability and its subsequent conversion to dopamine in the brain (Level 2) [28]. Several clinical trials have assessed the effects of MGBA modifications on levodopa pharmacokinetics, as summarized in [38]. Clinical trials show that *H. pylori* eradication provides a direct pharmacokinetic benefit, increasing levodopa AUC by approximately 20% and improving motor fluctuations (Level 1, interventional human trial) [28]. In an open-label trial of SIBO, bacterial eradication with rifaximin improved motor outcomes and reduced “OFF” time in 80% of patients, although without significant shifts in standard pharmacokinetic parameters, suggesting that the benefit may relate partly to transit time restoration rather than to exposure alone (Level 1/2, open-label, unblinded) [29].

Human trials of probiotics and prebiotics as adjuncts to levodopa/carbidopa treatment have also demonstrated clinically meaningful outcomes (Level 1/2) [38]. Administration of *Bifidobacterium animalis* subsp. *lactis* (Probio-M8) significantly improved Parkinson’s disease motor scores and increased serum dopamine levels in patients [52]. Similarly, administration of *Lactobacillus plantarum* PS128 resulted in improved motor scores and reduced “OFF” time in patients with Parkinson’s disease [53]. Prebiotic interventions, such as 10 g of daily fiber, reduced plasma zonulin (a marker of “leaky gut”) and improved motor function, suggesting that stabilizing the intestinal barrier is a viable clinical target [54]. Finally, fecal microbiota transplantation (FMT) in animal models induced a ~2.5-fold AUC surge and a 3–4-fold increase in striatal dopamine (Level 3, animal; human FMT trials for this indication are not yet available) [17].

The *H. pylori* eradication effect (~20% AUC increase) and the rifaximin/SIBO data are the most clinically mature findings in this review; both derive from human interventional studies, are of a magnitude relevant to dose titration, and are already actionable using existing, approved diagnostic and therapeutic tools (urea breath test/hydrogen breath test, standard eradication regimens). By contrast, AFMT and FMT-based interventions, while mechanistically compelling and quantitatively large in animal models (AFMT: C_max_ +50%, and FMT: AUC +150%), remain preclinical and are not presently available for human use.

Collectively, these data indicate the clinical importance of pre-systemic levodopa metabolism in the gut lumen, and support the gut microbiota as a target for modulation in the clinical management of patients with Parkinson’s disease to enhance and stabilize the therapeutic response to levodopa/carbidopa [20,21].

### 6.2. Olanzapine

#### 6.2.1. Pre-Systemic Metabolism of Olanzapine

Following oral administration, olanzapine undergoes extensive interaction with the gut microbiome (see Supplementary Table S3). In rat models, antibiotic-induced microbiota depletion precipitates a 1.8-fold increase in the systemic bioavailability of olanzapine after a single oral dose (Level 3, single rodent study, not yet independently replicated) [19]. The underlying mechanism for this surge, identified in rodent and in vitro systems, is the significant downregulation of intestinal UGT1A3 mRNA (five-fold reduction; the rat functional equivalent of human UGT1A4), the primary enzyme responsible for olanzapine glucuronidation, while the expression of major hepatic CYP450 enzymes involved in olanzapine metabolism remains largely unchanged (Level 3/4) [19]. These rat findings should be extrapolated to humans with caution. In humans, oral olanzapine bioavailability is approximately 60%, and its first-pass loss is attributed predominantly to hepatic metabolism (UGT1A4-mediated N-glucuronidation and CYP1A2-mediated oxidation); the relative contribution of intestinal UGT activity, and its dependence on the microbiome, has not been quantified in humans. In addition, the profound microbiota depletion achieved with antibiotic cocktails in rodents is likely greater than that produced by typical clinical antibiotic courses. Whether antibiotic exposure changes olanzapine exposure in patients is therefore unknown (Level 5 for human translation): no human PK study has addressed this question, and no clinical recommendation (e.g., dose adjustment or therapeutic drug monitoring after antibiotic treatment) can be derived from the current evidence. Appropriately designed clinical PK studies in patients receiving olanzapine with and without concomitant antibiotics are needed to test this hypothesis.

#### 6.2.2. The Obesogenic Transition and Metabolic Dysregulation

Olanzapine, together with clozapine, has among the highest propensities among atypical antipsychotics for inducing metabolic dysregulation [15], a side effect that, according to rodent data, is mediated in part by drug–gut–microbiome interaction [24]. In rodents, chronic olanzapine administration induces a rapid change in the microbiome termed the “obesogenic transition” (Level 3) [24,55], characterized by a five-fold increase in the *Firmicutes*: *Bacteroidetes* ratio and a marked depletion of commensal taxa such as *Blautia*, *Lachnospiraceae*, and *Parasutterella* [24].

The quantitative impact of these shifts on host physiology is substantial in animal models: a 30% increase in body weight, a 3-fold increase in blood glucose, and a 2.0–2.9-fold increase in plasma triglycerides (Level 3) [24,55]. Human cohort data corroborate the phenomenology, though not necessarily the microbiome-mediated mechanism, of this metabolic toxicity, with meta-analyses showing that a substantial proportion of patients experience >7% weight gain within the first year of treatment, alongside elevated fasting glucose and triglycerides (Level 2, human observational—association between olanzapine use and metabolic outcome, not a direct demonstration that the microbiome mediates the human effect) [15]. This drug-induced dysbiotic state in animals is further characterized by a > two-fold decrease in cecal short-chain fatty acids (SCFAs), particularly butyrate and propionate, which are essential for maintaining the integrity of the BBB (Level 3) [24,56].

#### 6.2.3. Causality, Transmission, and Therapeutic Modifiers

The mechanistic importance of the microbiome for olanzapine-mediated toxicity is supported by high-quality causal animal evidence: germ-free mice are almost entirely protected from olanzapine-induced weight gain, and the metabolic phenotype is partially transmissible via fecal microbiota transplantation (FMT) from olanzapine-treated to untreated animals (Level 3, strong causal evidence within the animal model) [57]. These findings satisfy the causality criteria outlined in Section 2.5 (directional perturbation plus phenotype transfer) more rigorously than most other mechanisms reviewed here, although the evidence remains confined to rodent models and has not been replicated in humans.

These mechanistic insights have led to the identification of specific therapeutic modifiers. Preclinical trials have evaluated prebiotic B-GOS (galacto-oligosaccharides) as a microbiome-targeted intervention. Co-administration of B-GOS with olanzapine in female rats successfully attenuated body weight gain and normalized plasma acetate levels without compromising CNS effects or treatment-induced changes in cortical expression of the 5-HT2AR serotonin receptor (a pharmacodynamic target adaptation) (Level 3) [38,56].

The olanzapine microbiome PK and metabolic toxicity literature is mechanistically rich and internally consistent across several independent rodent studies, but no human interventional or even human observational study has directly linked microbiome status to olanzapine exposure or metabolic outcome; the clinical relevance of this archetype should therefore be regarded as biologically plausible and mechanistically well-supported in animals, but presently unconfirmed and not actionable in human dosing decisions. Until confirmatory human data become available, the possibility that microbiome alterations (e.g., induced by antibiotics or probiotics) modify olanzapine exposure or its metabolic adverse effects should be regarded as a research hypothesis that does not justify changes in clinical dosing [19,24].

### 6.3. Sertraline

#### 6.3.1. Antimicrobial Effects vs. Sequestration and Metabolism in the Gut Lumen

Sertraline, and the selective serotonin reuptake inhibitor (SSRI) class more broadly, exemplifies a complex bidirectional interaction in which the drug functions as a potent non-antibiotic antimicrobial while its own efficacy is affected by the resulting microbial environment [22] (see Supplementary Table S3).

Large-scale in vitro screens and human fecal suspension assays demonstrate that sertraline possesses substantial antimicrobial activity, successfully suppressing the growth of approximately 73% of tested human gut bacterial strains (Level 4, in vitro/ex vivo) [13]. At the concentrations tested in vitro, which may differ from those reached in the colon of treated patients, this antimicrobial pressure could favor the loss of microbial diversity; whether it occurs in patients and contributes to the gastrointestinal adverse effects of sertraline has not been established (Level 5 for causality). Among other SSRIs, sertraline has both the highest antimicrobial activity and the strongest association with microscopic colitis and chronic diarrhea, likely reflecting a combination of microbial depletion with direct enteric serotonergic stimulation induced by the local effects of the drug on the enteric nervous system (Level 2, clinical association) [11].

Sertraline can, in turn, be affected by the gut microbiota through microbial sequestration and/or degradation. In ex vivo incubations with human fecal suspensions, sertraline concentrations declined by 20% to 80% within 48 h in the presence of certain microbial communities (Level 4) [10]. This figure should not be read as a 20–80% reduction in human oral bioavailability, for several reasons: (i) the incubations were performed in vitro under batch conditions with a 48 h duration, whereas sertraline, a lipophilic weak base, is largely absorbed in the proximal small intestine, where bacterial density is low and transit time is only a few hours; (ii) fecal suspensions represent the distal colonic community rather than the proximal small intestine; (iii) the depletion measured in vitro reflects disappearance of the parent drug from the medium by sequestration and/or metabolism (not distinguished), not a loss of absorbable drug in vivo; and (iv) sertraline undergoes extensive hepatic first-pass metabolism, the contribution of which to PK variability is likely to be much larger. Whether microbial depletion contributes to the PK variability of sertraline in patients is therefore unknown (Level 5 for clinical PK relevance), and this question requires integrated human PK/microbiome studies with sampling of the proximal small intestine (see Section 7.3).

#### 6.3.2. Pharmacodynamic Blockade

The potentially most clinically relevant aspect of sertraline’s interaction with the MGBA lies in the pharmacodynamic domain, where the microbiome shapes the biological context of the antidepressant response. Preclinical data from double-hit stress models in rodents show that a pro-inflammatory microbiota, characterized by low SCFA production and high lipopolysaccharide translocation, can almost completely block the efficacy of sertraline treatment (Level 3, animal, causal within the model) [36]. Importantly, and as flagged in Section 1.5, this specific double-hit stress paradigm was conducted with fluoxetine, not sertraline. It is cited here as class-level (SSRI) preclinical evidence for the general principle that pro-inflammatory dysbiosis can attenuate SSRI pharmacodynamic response, rather than as sertraline-specific data. Direct replication of this blockade with sertraline itself has not yet been reported, and this cross-drug extrapolation should be regarded as Level 3 for the SSRI class in general, but Level 5 (hypothetical) specifically for sertraline until a sertraline-specific study is available.

This blockade is mechanistically linked to activation of the kynurenine pathway, in which systemic inflammation shunts tryptophan away from serotonin synthesis and toward neurotoxic metabolites such as quinolinic acid (Level 3/4) [9]. This finding is clinically important because it suggests that resistance to sertraline treatment in humans may, in some patients, reflect a failure of the biological background rather than insufficient drug bioavailability or brain delivery. This interpretation itself remains associative in humans (Section 2.5) until tested interventionally (e.g., via a trial in which baseline inflammatory/dysbiotic status prospectively predicts sertraline response).

#### 6.3.3. Sertraline Clinical Trial Outcomes

Clinical evidence links successful therapeutic outcomes with the restoration of MGBA equilibrium. In human psychiatric cohorts, clinical recovery from a depressive episode is associated with a quantifiable normalization of the microbiome, specifically a ~1.12-fold increase in the Shannon index and a ~1.31-fold increase in the inverse Simpson index (Level 2, human observational, associative—see Section 2.5, since this cross-sectional design cannot establish whether diversity restoration is a cause or a consequence of clinical recovery) [58].

Furthermore, clinical trials of adjunctive microbiome-targeted strategies provide limited human interventional evidence that adjunctive psychobiotics may improve depressive symptoms in patients with major depressive disorder. Whether this reflects reversal of the proposed microbiome-dependent pharmacodynamic blockade of SSRIs has not been demonstrated, and these trials were not designed to test interactions with a specific SSRI such as sertraline. Trials using *Lactobacillus plantarum* 299v demonstrated a significant reduction in kynurenine concentrations and a concomitant improvement in cognitive function among patients with major depressive disorder (Level 1/2, depending on individual trial design) [59]. Other studies involving *Lactobacillus plantarum* PS128 and *Bifidobacterium longum* have shown modest but clinically meaningful improvements in depression and anxiety scores, likely by normalizing neuroimmune signaling and reducing the neurotoxic inflammatory burden (Level 1/2) [60,61,62,63].

The antimicrobial and sequestration data (Section 6.3.1) are robust in vitro findings with high internal reproducibility across strain panels, but their translation into human PK variability is untested. The pharmacodynamic blockade concept (Section 6.3.2) is mechanistically the most important sertraline MGBA finding in this review, but its evidentiary basis for sertraline specifically is currently indirect (extrapolated from fluoxetine); adjunctive psychobiotic trials (Section 6.3.3) represent the most immediately clinically feasible intervention, being low-risk, already marketed products, though effect sizes to date are modest and study quality is heterogeneous. Monitoring gut microbiome status and preventing and treating pro-inflammatory dysbiosis are reasonable, low-risk adjunctive considerations for treatment-resistant patients on sertraline and other SSRIs, but are not yet the standard of care.

### 6.4. Amitriptyline

#### 6.4.1. Antimicrobial Activity on the Gut Microbiome

Large-scale in vitro screens indicate that amitriptyline, alongside other tricyclic antidepressants, possesses substantial non-antibiotic antimicrobial activity, successfully suppressing the growth of approximately 73% of tested human gut bacterial strains (Level 4) (see Supplementary Table S3). This antimicrobial pressure produces a quantifiable reduction in microbial richness during chronic treatment of patients with amitriptyline (Level 2) [22]. Preclinical data in rat models of chronic unpredictable mild stress further characterize this shift; six weeks of oral amitriptyline treatment significantly altered the taxonomic landscape, decreasing the *Firmicutes*: *Bacteroidetes* ratio via expansion of *Bacteroides* and *Alistipes* (Level 3) [6]. These structural perturbations are accompanied by functional shifts in metagenomic pathways involved in carbohydrate metabolism, membrane transport, and signal transduction, suggesting that the drug reshapes the microbiome’s fundamental metabolic capacity (Level 3) [6].

#### 6.4.2. The Motility–Metabolic Feedback Loop

As established in both rodent and ex vivo models, amitriptyline’s potent anticholinergic activity significantly slows gastric emptying and intestinal transit (Level 3/4) [22,35]. While this effect is often viewed as a reason for side effects, such as constipation, it has profound biopharmaceutical implications. Slowed transit increases the luminal residence time of the gut lumen contents, providing a larger temporal window for microbial drug sequestration or metabolic degradation by bacterial enzymes (Level 5, mechanistic hypothesis, not directly quantified in a dedicated PK study) [22]. This creates a proposed self-reinforcing negative loop in which the drug’s primary enteric effect (dysmotility) amplifies its own pre-systemic loss due to the gut microbiome, potentially contributing to the well-recognized clinical variability in amitriptyline systemic exposure and clinical efficacy [6,22].

#### 6.4.3. Effects on Antibiotic Resistance in the Gut Microbiome

Beyond structural shifts in the gut microbiome, chronic amitriptyline administration exerts a distinct selective pressure on antibiotic resistance in the gut microbiome. In rat models of chronic unpredictable mild stress, long-term amitriptyline exposure significantly increased the abundance of genes conferring resistance to aminoglycosides and multidrug efflux pumps (Level 3, single animal study) [6]. This finding suggests that orally administered drugs with antimicrobial properties may inadvertently contribute to antimicrobial resistance within the host’s own microbiota (Level 3) [6]. While this mechanism is demonstrated in animal models, its direct clinical relevance to human antimicrobial resistance remains unclear and unconfirmed (Level 5) and requires assessment in prospective clinical trials.

#### 6.4.4. Amitriptyline Clinical Trial Outcomes

The clinical significance of amitriptyline’s interaction with the MGBA is best established in the context of irritable bowel syndrome, although the nature of the underlying evidence requires careful qualification. The landmark ATLANTIS trial provides high-quality human evidence of amitriptyline’s clinical efficacy in a disorder of gut–brain interaction (Level 1) [34,35], but it evaluated symptomatic benefit in irritable bowel syndrome without directly measuring gut microbiota composition, microbial metabolites, or pharmacokinetic parameters; accordingly, it constitutes indirect, supportive evidence for a drug–MGBA interaction rather than a direct demonstration of microbiome-mediated causation. In this randomized, double-blind, placebo-controlled phase 3 trial, low-dose amitriptyline (titrated up to 30 mg) significantly improved clinical symptom severity, reducing Irritable Bowel Syndrome Severity Scoring System scores by 23.3 points at three months (95% CI 4.6–42.0) and 27.0 points at six months (95% CI 7.1–46.9) compared with placebo; amitriptyline-treated patients were also more likely to report symptom relief on subjective global assessment (odds ratio 1.78, 95% CI 1.19–2.66) [34,35]. This is, notably, the single strongest piece of Level 1 human evidence supporting any drug–MGBA interaction reviewed in this manuscript, but attributing the observed therapeutic benefit directly to microbiome modulation remains an indirect inference: the ATLANTIS trial demonstrates clinical efficacy in a gut–brain axis disorder rather than directly measuring a microbiome-mediated mechanism. The mechanistic contribution of microbiome-mediated effects *per se* (distinct from direct CNS neuromodulation that attenuates the perception of visceral pain and discomfort, and from anticholinergic gut motility effects that may ameliorate diarrhea-predominant irritable bowel syndrome symptoms) remains inferential and should not be described as established causal evidence.

Amitriptyline exemplifies the “mixed mechanism” archetype well: the microbiome-remodeling and resistome data are robust but purely preclinical (Level 3/4), while the clinical outcome data (ATLANTIS) are the highest-quality (Level 1) evidence in this review, yet address a clinical endpoint (IBS symptom severity) rather than a directly measured microbiome–PK/PD linkage. Clinicians can act today on the ATLANTIS efficacy data; the mechanistic microbiome contribution to that efficacy, and the proposed motility–metabolic feedback loop, remain research targets rather than actionable clinical parameters.

Collectively, these data indicate that, for amitriptyline, a delicate balance must be managed in individual patients between the well-established clinical benefit in disorders of gut–brain interaction and the theoretical potential for drug-induced dysmotility to exacerbate microbial drug loss and long-term ecological remodeling of the gut microbiome [38].

## 7. Discussion

### 7.1. Mechanism and Location of Clinically Important MGBA–Drug Interactions

The evidence synthesized in this review establishes the MGBA as an important non-passive regulator of CNS pharmacotherapy [2]. As illustrated in the bidirectional communication pathways of Figure 1, the intestinal microbiome and its metabolites affect biopharmaceutical and pharmacokinetic processes that alter systemic drug exposure while simultaneously affecting the pharmacodynamic processes in the CNS and elsewhere [3,22]. The gut can no longer be viewed as a passive absorption site; it is an active metabolic organ capable of shifting the entire coupled steady state of the MGBA–CNS system [5,41].

The mechanism and location of clinically important interactions differ among the four archetype drugs introduced in Section 1.5 (see Table 1, which summarizes the principal findings, the human and causal evidence, and the current actionability for each of the four archetypal drugs, with more detailed descriptions provided in Tables S1–S4). For levodopa/carbidopa, the interaction is mostly biopharmaceutical, governed by pre-systemic metabolism of levodopa by gut bacteria, which is inefficiently blocked by carbidopa (see Section 6.1). For olanzapine, systemic bioavailability is substantially affected by the gut microbiome, and microbiota depletion reduces pre-systemic drug loss and enhances the drug’s systemic exposure (see Section 6.2). For sertraline, pharmacodynamic factors in the CNS appear to be predominant, with pro-inflammatory dysbiosis interfering with the therapeutic efficacy of the drug via the kynurenine pathway (as detailed in Section 6.3). For amitriptyline, no single mechanism or location predominates; rather, a combination of the biopharmaceutical, pharmacokinetic, and pharmacodynamic factors (drug–microbiome interaction in the lumen, effects on the GI motility, and CNS neuromodulation; see Section 6.4).

### 7.2. The Microbiome-Dependent Pharmacological Phenotype

The data reviewed here converge on the concept that an individual patient’s gut microbiome constitutes a distinct, partially modifiable determinant of CNS drug response, one that can be described as a microbiome-dependent pharmacological phenotype (MDPP). We propose that this phenotype can be conceptually decomposed into five interacting components, each of which has been illustrated by at least one of the four archetype drugs reviewed in Section 6:

1. Microbial enzymatic capacity—the abundance and activity of drug-metabolizing bacterial enzymes in an individual’s gut lumen (e.g., fecal *tdc* abundance for levodopa; strain-level antimicrobial susceptibility for sertraline and amitriptyline).
2. Metabolite production profile—the individual’s capacity to generate SCFAs, kynurenine pathway metabolites, secondary bile acids, and other MGBA-relevant signaling molecules, which shapes both drug disposition (e.g., pH-dependent solubility) and target–organ context (e.g., BBB integrity, neuroinflammatory tone).
3. Intestinal barrier status—the degree of paracellular permeability (“leaky gut”), which determines both passive drug/metabolite absorption and the systemic translocation of pro-inflammatory bacterial products.
4. Systemic inflammatory state—the downstream consequence of barrier status and metabolite profile, which feeds back to alter hepatic and CNS drug-target pharmacology (e.g., kynurenine pathway activation blunting SSRI response).
5. Drug exposure variability—the net pharmacokinetic consequence of components one to three in combination with classical host covariates (age, sex, hepatic/renal function, pharmacogenetics).

Within this framework, a given patient’s MDPP could, in principle, help explain why two individuals receiving identical doses of the same CNS drug, with similar classical covariates, nonetheless show divergent efficacy or toxicity. For levodopa, high microbial *tdc* enzymatic capacity (component one) has the best-established quantitative link to dose requirement (Section 6.1.1); for sertraline, an individual’s inflammatory/dysbiotic state (components three to four) may better predict pharmacodynamic response than plasma drug concentration (Section 6.3.2); for olanzapine, all five components are mechanistically plausible contributors but remain characterized only in animal models (Section 6.2).

We emphasize that the MDPP concept, as presented here, is an organizing framework for future hypothesis-driven research and biomarker development, not a validated clinical tool; no prospective study has yet measured all five components simultaneously in a single patient cohort and linked the composite phenotype to a CNS drug outcome. Its principal value at present is to reframe “unexplained” interindividual pharmacological variability as a set of measurable, and potentially modifiable, microbiome-related parameters, providing a concrete research agenda for the biomarker panels discussed in Section 7.3.

### 7.3. Methodological Gaps and the Need for Standardized Biomarkers

Despite extensive data on MGBA–drug interactions in preclinical systems (in vitro, ex vivo, animal studies), their clinical translation is not straightforward. In many studies, relative microbial abundance is quantified, rather than the absolute amounts of specific bacterial strains needed to assess the microbial load and its impact on drug metabolism [38]. Furthermore, stool samples, which are frequently used as a source of information on the gut microbiome, often fail to reflect the small intestinal microbiome, which is the major anatomical location of the biopharmaceutical and pharmacokinetic aspects of MGBA–drug interactions (pre-systemic drug sequestration and metabolism, drug absorption with concomitant first-pass metabolism; see Section 3 and Section 4).

To address these methodological gaps, future MGBA–CNS drug studies should adopt concrete experimental and clinical designs that directly sample the biopharmaceutically relevant gut segments and collect mechanistically informative biomarkers. First, targeted sampling of the small intestine is essential, since most pre-systemic drug–microbiome interactions occur in the duodenum and jejunum rather than the colon. Jejunal aspirate sampling (via endoscopy) and capsule-based biopsy/aspiration systems can be used to obtain luminal contents and mucosal biopsies from the proximal small intestine at defined time points relative to drug administration, enabling quantification of bacterial gene abundance (e.g., *tdc* for levodopa, microbial β-glucuronidases for enterohepatic cycling) and measurement of local drug concentrations, metabolites, pH, and SCFA profiles at the actual absorption site, rather than relying solely on stool as a distal surrogate.

In parallel, non-invasive or minimally invasive biospecimens should be standardized. Stool samples remain valuable for global taxonomic profiling and fecal metabolomics, but should be complemented by blood-based markers of barrier integrity (e.g., zonulin, S100B), systemic inflammation (e.g., IL-6, TNF-α, high-sensitivity CRP), and metabolic signatures (e.g., SCFAs, kynurenine/tryptophan ratio). When feasible, cerebrospinal fluid or advanced neuroimaging readouts (e.g., PET or MRI measures of neuroinflammation, BDNF-related plasticity) can provide direct CNS pharmacodynamic endpoints, though these will be restricted to specialized settings.

### 7.4. Limitations and Translational Barriers

Several important limitations qualify the conclusions of this review and constrain the near-term clinical translation of MGBA-informed CNS pharmacotherapy:

Review methodology. As detailed in Section 2.1, this is a narrative review conducted and screened by a single author, without independent dual review, formal PRISMA reporting, or a pre-registered protocol. This design is appropriate for a mechanistically oriented critical review but carries an inherent risk of selection and confirmation bias, particularly given that the underlying primary literature itself is disproportionately composed of positive, mechanistically confirmatory findings (a form of publication bias common in an emerging field); negative or null microbiome–drug studies are almost certainly underrepresented in the sources available for citation. In addition, the search relied partly on general Google searches, which are not fully reproducible, and the 64 cited records reflect a selective scope; the review should therefore not be considered exhaustive or a substitute for a systematic review.

Interindividual and geographic variability of the microbiome. Human gut microbiome composition varies substantially with geography, diet, host genetics, and age, independent of any drug exposure. Nearly all of the quantitative effect sizes cited in this review derive from single-cohort or single-laboratory studies (frequently in Western populations or in a single rodent facility), and the extent to which findings generalize across populations with different baseline microbiome compositions is unknown.

Dietary and geographical confounding. Diet is both a major determinant of microbiome composition and a frequent, incompletely controlled confounder in the clinical studies cited here (Section on lifestyle/nutritional modifiers, Supplementary Table S2); disentangling direct drug–microbiome effects from diet-driven microbiome variation will require dietary standardization or covariate adjustment in future trials.

Animal-to-human translational gap. As emphasized throughout Section 3, Section 4, Section 5 and Section 6, a large majority of the mechanistic and quantitative data reviewed here (most olanzapine data; much of the levodopa secondary-metabolism and amitriptyline resistome data) derive exclusively from rodent models. Mice and rats differ from humans in gut microbial composition, gut transit time, and relevant enzyme orthologs (e.g., rat UGT1A3 as a surrogate for human UGT1A4), and effect sizes measured in animals should not be assumed to translate quantitatively, or even directionally, to humans.

Absence of validated microbiome biomarkers for routine pharmacological use. No microbiome-derived biomarker discussed in this review (fecal *tdc* abundance, strain-level antimicrobial susceptibility, SCFA panels, zonulin) has been validated as a companion diagnostic or incorporated into a regulator-approved dosing algorithm. Assay standardization (sampling site, storage, sequencing/qPCR platform, reference ranges) remains an unmet need and a prerequisite for any of the biomarker panels proposed in Section 7.3.

Standardization and reproducibility of microbiome methodology. Differences in DNA extraction, sequencing depth, bioinformatic pipelines, and the use of relative versus absolute abundance measures (Section 7.3) limit cross-study comparability and are a further barrier to clinical implementation.

Taken together, these limitations mean that, of the interactions reviewed here, only a small subset (most clearly, *H. pylori* eradication and SIBO treatment for levodopa, see Section 6.1.3, and low-dose amitriptyline for IBS, see Section 6.4.4) are supported by human interventional evidence sufficient to inform current clinical practice; the remainder should be regarded as emerging, mechanistically plausible, or hypothesis-generating, as summarized by the evidence tiers assigned throughout Section 3, Section 4, Section 5 and Section 6 and in Supplementary Tables S1–S4.

### 7.5. Shifting the Pharmacometric Paradigm: Host–Microbiome Modeling

Based on the data summarized in this review, while bearing in mind the limitations described in Section 7.4, it appears that host-centric pharmacokinetic models that do not account for MGBA–drug interactions may inadequately describe the biofate of orally administered CNS drugs [5]. We argue for the integration of a gut–microbiome compartment into mechanistic PK/PD models. For this purpose, in clinical trials, each patient could be represented by a joint data structure comprising: (a) a systemic PK profile for the drug and relevant metabolites (plasma concentrations over time, AUC, C_max_, clearance, and volume of distribution); (b) a microbiome and luminal environment profile (taxa, gene abundance, SCFAs, pH, motility indices) at baseline and at predefined on-treatment time points; and (c) a host response profile (barrier integrity markers, cytokines, kynurenine/tryptophan ratio, BDNF, cortisol, and clinical outcome scores), corresponding to the five MDPP components introduced in Section 7.2.

These domains can be linked in several complementary ways. At the statistical level, mixed-effects PK/PD models can include microbiome-related covariates (e.g., *tdc* abundance, SCFA concentrations, transit time, zonulin levels) to explain interindividual variability in exposure (AUC, C_max_) and response (clinical scores), formally quantifying the contribution of each MGBA factor to PK and PD variability. At the mechanistic level, physiologically-based pharmacokinetic (PBPK) models can incorporate a gut–microbiome compartment with parameters representing luminal clearance by microbial metabolism and bioaccumulation, pH-dependent solubility, motility-driven residence time, and microbiome-modulated permeability. Biomarkers such as *tdc* gene copy numbers, SCFA concentrations, and zonulin levels could then parameterize or calibrate this compartment for individual patients or subgroups.

Finally, longitudinal biomarker measurements would allow assessment of how therapeutic MGBA modifiers (e.g., antibiotics, PPIs, probiotics, prebiotics, FMT) shift the microbiome and barrier state over time, and how these shifts are reflected in corresponding changes in PK (exposure) and PD (clinical effect size, tolerability). Such integrated designs are needed to move from descriptive association toward quantitative, causal models in which MGBA-related biomarkers can be used prospectively for patient stratification, dose optimization, and rational selection of CNS drugs or adjunctive microbiome-targeted therapies.

### 7.6. MGBA–Drug Interactions and Personalized Medicine

The data synthesized in this review indicate that MGBA–drug interactions introduce an additional, highly individualized layer of variability into CNS pharmacotherapy, beyond classical host-centric factors such as age, sex, organ function, and pharmacogenetics. The MDPP framework proposed in Section 7.2 offers one way to organize this variability conceptually. Baseline microbial composition and metabolic capacity can determine whether a given patient experiences therapeutic benefit, suboptimal response, or toxicity with a specific orally administered CNS drug, and patients with greater microbial capacity for pre-systemic drug metabolism or sequestration may paradoxically require higher doses or exhibit apparent treatment resistance, despite adherence and apparently adequate systemic exposure.

From a personalized medicine perspective, MGBA factors should be regarded as modifiable covariates that could eventually be incorporated, alongside host genetics and clinical characteristics, into the selection and optimization of CNS therapies. In practical terms, this would involve stratifying patients using microbial gene abundance (e.g., *tdc* for levodopa), fecal SCFA profiles, kynurenine/tryptophan ratios, and barrier integrity markers, and using this information to inform drug choice, dosage, and the use of adjunctive microbiome-targeted interventions (such as probiotics, prebiotics, synbiotics, fecal microbiota transplantation, or antibiotics) [64].

However, as emphasized in Section 7.4, routine clinical implementation of MGBA-informed personalized CNS therapy remains aspirational at present, and this distinction between current clinical applications, emerging translational opportunities, and future research directions should be kept explicit. Most available data derive from preclinical models, small observational cohorts, or proof-of-concept trials, and standardized biomarker panels, validated companion diagnostics, and regulatory frameworks are still lacking. Before MGBA-related parameters can be implemented in routine dose individualization or therapeutic selection, prospective clinical studies must establish that microbiome-guided strategies lead to meaningful improvements in clinical outcomes and that the associated analytical assays are robust, reproducible, and economically feasible. In the interim stage, the primary implication for clinicians and clinical pharmacologists is awareness: MGBA perturbations, whether from underlying disease, diet, or comedications such as antibiotics, PPIs, and anti-inflammatory drugs, should be recognized as potential contributors to unexplained variability in CNS drug exposure and response (see Supplementary Table S4), even before validated biomarkers or dosing algorithms exist.

## 8. Conclusions

This critical mechanistic review demonstrates that the gut microbiome is a quantifiable, bidirectional modifier of CNS drug biofate, and that the four archetype drugs analyzed here (levodopa/carbidopa, olanzapine, sertraline, and amitriptyline) illustrate distinct, mechanistically well-defined routes (biopharmaceutical, pharmacokinetic, pharmacodynamic, and mixed, respectively) through which the MGBA can shape drug exposure and clinical response. Applying the five-tier evidence-grading framework introduced in Section 2.4 across this literature reveals a wide and clinically important spread in evidentiary maturity: at one end, *H. pylori* eradication and SIBO treatment for levodopa, and low-dose amitriptyline for IBS (the ATLANTIS trial), are supported by Level 1/Level 2 human interventional or observational data and are, to varying degrees, already actionable with existing clinical tools; at the other end, the olanzapine bioavailability and obesogenic-transition literature, and much of the sertraline pharmacodynamic blockade mechanism, remain confined to Level 3/Level 4 animal and in vitro evidence, with human translation still hypothetical.

Currently, only a small number of MGBA-informed interventions, including *H. pylori*/SIBO management in Parkinson’s disease, and amitriptyline for disorders of gut–brain interaction, can be considered part of existing clinical practice, albeit typically without explicit recognition of their microbiome-mediated component. Adjunctive psychobiotic, prebiotic, and dietary interventions represent the nearest-term emerging translational opportunities, being low-risk and already available, but currently supported mainly by modest-effect-size heterogeneous human trials. The remaining interactions reviewed here, including the microbiome-dependent pharmacological phenotype framework proposed in Section 7.2, and the PBPK/PD modeling strategy proposed in Section 7.5, constitute a research agenda rather than a set of present-day clinical tools.

Realizing the translational potential of this field will require: (i) prospective, ideally multi-site and dual-reviewer-conducted clinical trials that integrate standardized MGBA biomarker panels (including small intestinal, not only fecal, sampling) with pharmacokinetic and pharmacodynamic endpoints; (ii) independent replication of key preclinical findings, particularly for olanzapine and for the sertraline-specific pharmacodynamic blockade currently inferred from fluoxetine data; (iii) pharmacokinetic–pharmacodynamic models that explicitly incorporate a microbiome compartment; and (iv) validated, standardized microbiome biomarker assays suitable for regulatory qualification as companion diagnostics. Achieving these goals will be necessary to transform the currently unpredictable interindividual variability in CNS drug therapy into quantitatively understood and clinically actionable differences, and will require close, sustained collaboration between neurologists, psychiatrists, gastroenterologists, microbiologists, and clinical pharmacologists.

## Figures and Tables

**Figure 1:**
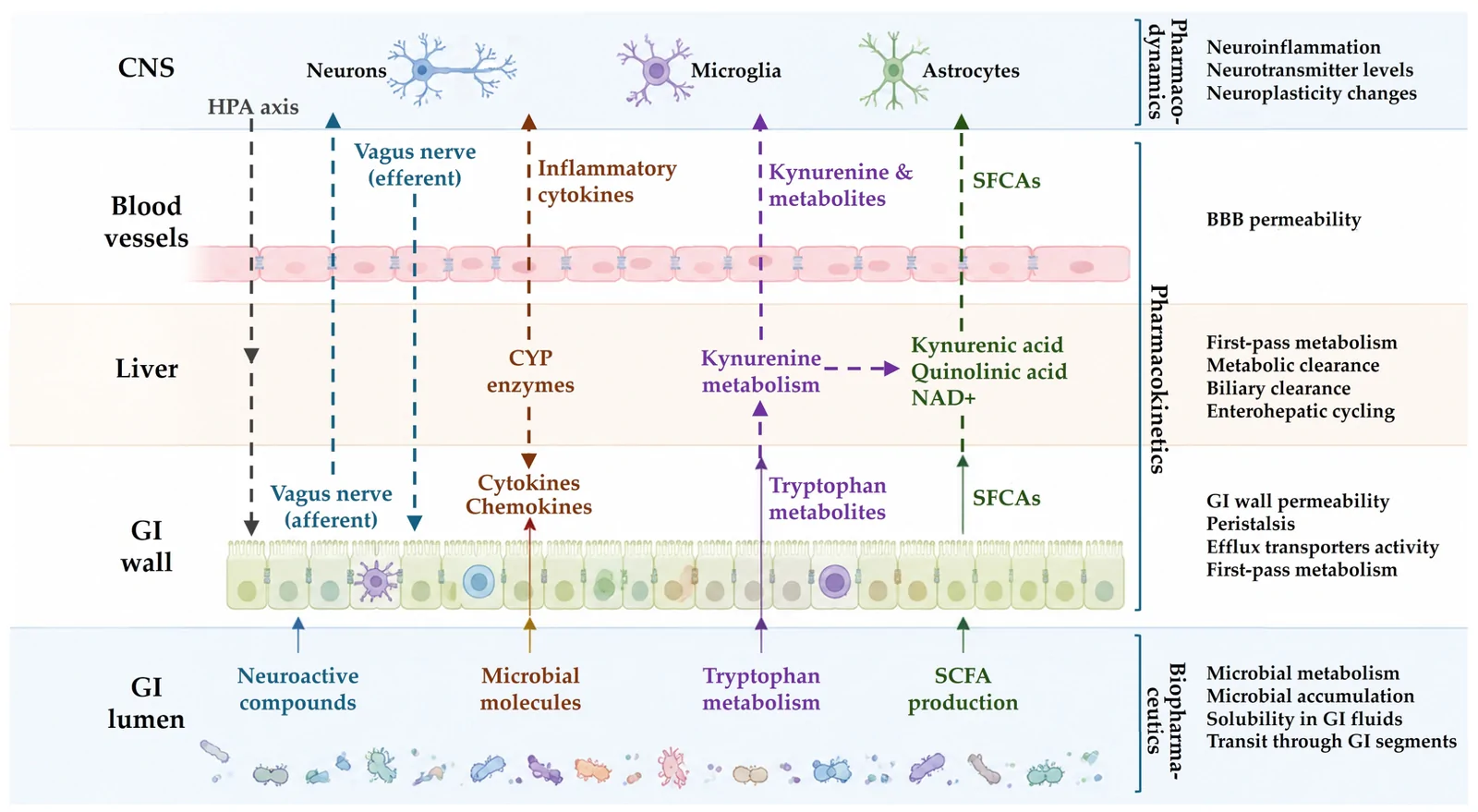
The bidirectional microbiota–gut–brain axis and its interactions with CNS drug biofate. For full details on the specific drugs, their tier assignments (L1–L5: human RCT, human observational, animal in vivo, in vitro/mechanistic or hypothetical), and corresponding citations, see Table S1.

**Table 1:** Summary of the principal MGBA–drug interactions for the four archetype drugs and the strength of the supporting evidence. Evidence tiers (L1–L5) are defined in Section 2.4; detailed data, individual studies, and references appear in Supplementary Tables S1–S4. “Human PK evidence” refers to studies in which drug exposure was measured in humans; “Causal evidence” refers to experimental manipulation of the microbiome or resolution of the molecular mechanism (Section 2.5).

Drug (Archetype)	Principal MGBA Mechanism and Magnitude	Human PK Evidence	Human Clinical/PD Evidence	Causal Evidence for a Microbiome Role	Strongest Tier and Current Actionability
Levodopa/carbidopa (biopharmaceutical-dominant)	Luminal decarboxylation by bacterial TDC; carbidopa 14,000–19,000-fold less potent against bacterial TDC than human AADC; up to 56% pre-systemic loss	*H. pylori* eradication: AUC ~+20% (L1); rifaximin for SIBO: no significant PK change (L1/L2)	Fecal *tdc* abundance correlates with daily dose (r = 0.66; L2, cross-sectional); motor improvement after *H. pylori* eradication or rifaximin	Enzyme-level mechanism (L4); microbiota depletion, FMT, and AFMT in rodents (L3/L4); in humans, associative for *tdc*	L1–L2. *H. pylori*/SIBO management actionable; *tdc* testing and microbiome-targeted therapy not yet actionable
Olanzapine (PK-dominant; metabolic toxicity)	Microbiota depletion: ~1.8-fold higher bioavailability in rats via lower intestinal UGT1A3 (single study); drug-induced “obesogenic transition” in rodents	None	Olanzapine-associated weight gain is established (>7% within one year); microbiome mediation not shown in humans (L2 for the phenomenon only)	Germ-free protection and FMT transfer of the weight-gain phenotype in mice (L3)	L3 (human translation L5). Not actionable
Sertraline (PD-dominant)	~73% of tested strains suppressed in vitro; 20–80% depletion in fecal suspensions at 48 h (not equivalent to reduced bioavailability); PD blockade by pro-inflammatory dysbiosis shown with fluoxetine	None	Diversity increases accompany recovery (L2, associative); adjunctive psychobiotic trials in MDD show modest, heterogeneous effects (L1/L2)	Fluoxetine-based rodent data (L3, SSRI class); no sertraline-specific data (L5)	L3–L4 for sertraline-specific mechanisms. Adjunctive psychobiotics low-risk but not standard of care
Amitriptyline (mixed mechanism)	~73% of tested strains suppressed in vitro; anticholinergic transit slowing; microbiome and resistome shifts in rodents	None related to the microbiome	ATLANTIS RCT in IBS: IBS-SSS reduced by 23.3 (3 months) and 27.0 points (6 months) vs. placebo (L1); microbiome not measured	None; microbiome contribution to clinical benefit is inferential	L1 for clinical efficacy only. Amitriptyline for IBS is actionable; its microbiome component is not

## Data Availability

No new data were created or analyzed in this study. Data sharing is not applicable to this article.

## Supplementary Materials

The following supporting information can be downloaded at https://www.mdpi.com/article/10.3390/pharmaceutics18101276/s1, Table S1: Mechanistic Framework of Microbiota–Gut–Brain Axis Interactions with CNS Drugs; Table S2: Clinical, Physiological, and Therapeutic Modifiers of Microbiota–Gut–Brain Axis Interactions with CNS Drugs; Table S3: Mechanisms and Quantitative Data on Microbiota–Gut–Brain Axis Interactions with Selected CNS drugs; Table S4: Interdisciplinary Management of CNS Drug Therapy: Clinical Perturbations, Therapeutic Modifiers, Mechanistic Drivers, and Cross-Specialty Action Points.

## Abbreviations

The following abbreviations are used in this manuscript:

ADME	Absorption, distribution, metabolism, and excretion
AUC	Area under the curve
B-GOS	Bimuno™ (galacto-oligosaccharides)
BBB	Blood–brain barrier
BDNF	Brain-derived neurotrophic factor
C_max_	Peak serum/plasma concentration
CNS	Central nervous system
CYP450	Cytochrome P450
FMT	Fecal microbiota transplant
GI	Gastrointestinal
HPA axis	Hypothalamic–pituitary–adrenal axis
IL-6	Interleukin-6
LPS	Lipopolysaccharides
MDPP	Microbiome-dependent pharmacological phenotype
MGBA	Microbiota–gut–brain axis
P-gp	P-glycoprotein
PD	Pharmacodynamic
PK	Pharmacokinetic
PPIs	Proton pump inhibitors
SCFAs	Short-chain fatty acids
SIBO	Small intestinal bacterial overgrowth
SSRI	Selective serotonin reuptake inhibitor
TCAs	Tricyclic antidepressants
TDC/*tdc*	Tyrosine decarboxylase
TNF-α	Tumor necrosis factor-alpha
UGT	Uridine diphosphate-glucuronosyltransferase
