# Supplementary material for: Microbiota–Gut–Brain Axis Interactions with CNS Drugs: A Critical Mechanistic Review of Biopharmaceutical, Pharmacokinetic, and Pharmacodynamic Evidence
Source: Pharmaceutics. 2026 Oct 9;18(10):1276. doi: 10.3390/pharmaceutics18101276 (PMC13708323; doi:10.3390/pharmaceutics18101276)
Supplement: Supplementary file 1 [file pharmaceutics-18-01276-s001.zip › pharmaceutics-4599680-supplementary.pdf]

Microbiota-Gut-Brain Axis Interactions with CNS Drugs: A Critical Mechanistic Review of Biopharmaceutical, Pharmacokinetic, and Pharmacodynamic Evidence

David Stepensky [1,*]

[1] Department of Clinical Biochemistry and Pharmacology, Faculty of Health Sciences, Ben-Gurion University of the Negev, Beer-Sheva 84105, Israel

* Correspondence: Department of Clinical Biochemistry and Pharmacology, Faculty of Health Sciences, Ben-Gurion University of the Negev, Beer-Sheva 84105, Israel, e-mail: davidst@bgu.ac.il, Tel.: +972-8-6477381

Evidence-Grading Legend (applies to Tables S1–S4)*

Level	Definition
L1	Human randomized or other controlled interventional trial
L2	Human observational, cohort, cross-sectional, or single-arm/open-label interventional study
L3	Animal (*in vivo*) model, including germ-free, gnotobiotic, antibiotic-depletion, and FMT rodent studies
L4	*In vitro* / *ex vivo* / mechanistic study using human- or animal-derived material outside a living host
L5	Hypothetical or not yet experimentally tested mechanism, proposed by analogy or pathway plausibility

* Levels are assigned to the strongest available evidence tier for each row;

Table S1. Mechanistic Framework of Microbiota-Gut-Brain Axis Interactions with CNS Drugs

Domain	Mechanistic Pathway	Core Biological Process	Major Mediators / Components	Pharmacological Consequence	Representative CNS Drug Examples	Evidence Level
Bidirectional gut-brain communication	Brain → gut signaling	CNS-mediated modulation of gut motility, secretion, permeability, and microbial ecology	Cortisol, autonomic nervous system, vagal efferents, enteric neurotransmission	CNS disease and stress alter oral drug absorption and microbial metabolism	Chronic stress altering antidepressant response; neurotrauma-induced dysbiosis	L3
	Gut → brain signaling	Gut-derived metabolites and inflammatory signals alter CNS function and target sensitivity	SCFAs, kynurenine metabolites, cytokines, LPS, bile acids	Alters neuroinflammation, BBB integrity, and drug responsiveness	Sertraline resistance in inflammatory microbiota	L3
Biopharmaceutical	Microbial metabolism	Enzymatic transformation of drugs before host absorption	Decarboxylases, dehydroxylases, reductases, deaminases	Drug inactivation, activation, or toxic metabolite generation	Levodopa decarboxylation by *Enterococcus faecalis*	L4, corroborated by L2
	Microbial bioaccumulation ("metabolic sink")	Intracellular sequestration of drugs without chemical transformation	Strain-dependent bacterial uptake systems	Reduced fraction available for absorption; altered luminal exposure time	Duloxetine accumulation in commensals; possible SSRI sequestration	L4
	Luminal contents physicochemical modulation	Alteration of pH, fluid composition, viscosity, and solubility	SCFAs, bile acids, microbial fermentation products	Alters dissolution and ionization of weak acids/bases	Levodopa instability; altered olanzapine dissolution	L4-L5
	Gastrointestinal motility modulation	Altered transit time changes duration of luminal microbial exposure	Enteric serotonin, acetylcholine, vagal signaling	Changes residence time available for microbial metabolism	Amitriptyline-induced slowing of GI transit; SSRI-associated diarrhea	L2-L3
	Intestinal barrier permeability ("leaky gut")	Tight-junction disruption alters paracellular transport	Zonulin, occludin, claudins, LPS, cytokines	Alters passive absorption and endotoxin translocation	Dysbiosis-associated variability in psychotropic exposure	L3-L4
	Reciprocal drug-on-microbiome effects	CNS drugs directly alter microbial ecology	Intrinsic antimicrobial activity of psychotropics	Drug reshapes the ecosystem that later determines	SSRIs and TCAs suppress numerous microbiota strains	L4

Domain	Mechanistic Pathway	Core Biological Process	Major Mediators / Components	Pharmacological Consequence	Representative CNS Drug Examples	Evidence Level
				efficacy/toxicity		
Pharmacokinetic	Intestinal first-pass metabolism modulation	Microbiome regulates intestinal metabolic enzymes	UGTs, CYP3A4, CYP1A2, nuclear receptor signaling	Alters oral bioavailability before hepatic entry	Olanzapine bioavailability increases ~1.8-fold after microbiota depletion	L3 (single rodent study)
	Hepatic metabolic regulation	Microbial metabolites regulate liver metabolic capacity	p-cresol, bile acids, inflammatory cytokines	Alters systemic clearance and metabolite formation	CYP modulation during dysbiosis	L5
	Enterohepatic circulation	Microbial deconjugation reactivates excreted metabolites	β-glucuronidase-producing taxa	Prolonged systemic exposure and altered half-life	Glucuronidated antipsychotics and antidepressants	L4
	Host transporter regulation	Microbial signals regulate drug influx/efflux systems	P-glycoprotein, Large Neutral Amino acid Transporter 1	Alters intestinal absorption and CNS penetration	LAT1 competition reducing Levodopa transport	L3-L4
	Blood-brain barrier (BBB) regulation	Microbiota determines BBB permeability and tight-junction integrity	SCFAs, trimethylamine N-oxide, cytokines, microglial signaling	Alters fraction of circulating drug reaching CNS	SCFA depletion impairing BBB integrity	L3
	Sex-dependent microbiome-PK interactions	Hormonal modulation of microbiota and host metabolism	Estrogens, androgens, CYP regulation	Sex-specific exposure variability	Female PD patients show ~2.1-fold higher Levodopa C_{max}	L2
	Polypharmacy-mediated microbiome remodeling	Non-CNS drugs alter microbiome and host metabolism	PPIs, antibiotics, NSAIDs, metformin	Secondary alteration of CNS drug PK	PPI-associated *Enterococcus* overgrowth reducing Levodopa exposure	L2
Pharmacodynamic	Neuroimmune signaling	Gut-derived inflammation alters CNS immune state	IL-6, TNFα, microglial activation, LPS	Reduced target sensitivity and altered therapeutic response	SSRI resistance in inflammatory states	L2, associative in humans
	Neurotransmitter precursor regulation	Microbiota modulates precursor availability for CNS	Tryptophan, tyrosine, kynurenine metabolites	Alters serotonergic and dopaminergic tone	Kynurenine pathway activation reducing SSRI	L3-L4

Domain	Mechanistic Pathway	Core Biological Process	Major Mediators / Components	Pharmacological Consequence	Representative CNS Drug Examples	Evidence Level
		neurotransmitters			responsiveness	
	Vagus nerve signaling	Direct neural gut-brain communication independent of systemic drug levels	Vagal afferents/efferents, neuropods	Rapid modulation of emotional and autonomic processing	Psychobiotic effects abolished after vagotomy	L3
	Neuroplasticity modulation	Microbial metabolites regulate neuronal growth and plasticity	BDNF, SCFAs, histone deacetylases inhibition	Alters long-term responsiveness to psychotropics	Butyrate-associated enhancement of antidepressant effects	L3
	HPA-axis modulation	Dysbiosis alters stress-axis tone and cortisol signaling	Cortisol, corticotropin-releasing hormone, adrenocorticotropic hormone	Reinforces chronic inflammatory and dysbiotic states	Stress-associated antidepressant resistance	L3
	Central receptor adaptation	Chronic dysbiosis modifies CNS receptor expression	5-HT2AR, dopamine receptors, inflammatory signaling	Alters CNS target sensitivity despite stable exposure	Olanzapine-associated reduction in cortical 5-HT2AR	L3
	Functional PD-context modulation	Microbiome determines whether therapeutic concentrations translate into clinical response	Inflammation, SCFA depletion, kynurenine activation	Adequate plasma levels may still produce treatment failure	Depressive microbiota abolishing SSRI response	L3, associative human correlate L2
Therapeutic / Translational MGBA Modulation	Psychobiotics and probiotics	Restoration of microbial diversity and metabolite production	*Lactobacillus*, *Bifidobacterium*, GABA signaling	Improves barrier function and antidepressant responsiveness	Adjunctive therapy in MDD and PD	L1-L2
	Prebiotics and postbiotics	Promotion or direct delivery of beneficial microbial metabolites	B-GOS, inulin, butyrate	Reinforces gut barrier and BBB integrity	Reduction of olanzapine-induced metabolic dysfunction	L3
	Fecal microbiota transplantation (FMT)	Ecosystem replacement and responder phenotype transfer	Whole microbial community transfer	Alters systemic exposure and CNS responsiveness	FMT increasing Levodopa AUC ~2.5-fold	L3 (animal model only)

Domain	Mechanistic Pathway	Core Biological Process	Major Mediators / Components	Pharmacological Consequence	Representative CNS Drug Examples	Evidence Level
	Precision microbial enzyme inhibition	Selective targeting of bacterial drug-metabolizing enzymes	AFMT inhibition of tyrosine decarboxylase	Improves bioavailability without affecting host metabolism	AFMT increasing Levodopa C_{max} ~1.5-fold	L4

Table S2. Clinical, Physiological, and Therapeutic Modifiers of Microbiota-Gut-Brain Axis Interactions with CNS Drugs

Category	Specific Factor / Perturbation	Dominant MGBA Mechanism(s)	Impact on CNS Drug PK/PD	Clinical / Translational Significance	Evidence Level	Source
Disease-Related Perturbations	Baseline dysbiosis	Reduced alpha diversity; depletion of SCFA-producing taxa (*Faecalibacterium*, *Roseburia*); enrichment of pro-inflammatory organisms	Creates pro-inflammatory PD background that attenuates antidepressant response despite adequate exposure	Important contributor to treatment-resistant depression and altered psychotropic sensitivity; associative, not yet causally proven in humans	L2	[1]
	Small intestinal bacterial overgrowth (SIBO)	Increased proximal bacterial density; enhanced luminal metabolism; intestinal inflammation	Intensifies premature Levodopa metabolism and absorption variability	Present in ~54–67% of PD patients; contributes to OFF periods and delayed ON; rifaximin treatment is a Level-1/2 actionable intervention	L1-L2	[2, 3]
	Helicobacter pylori infection	Delayed gastric emptying; altered pH; inflammatory disruption of proximal absorption	Reduces Levodopa bioavailability and causes erratic absorption	Eradication improves symptomatic ON-time	L1	[4, 5]
	Inflammatory bowel disease (IBD)	Cytokine activation; barrier disruption; increased permeability; neuroimmune signaling	Alters oral absorption and increases neuroinflammatory burden	Associated with 30–40% prevalence of anxiety/depression; (association, not proven causal direction)	L2	[6, 7]
	Neurotrauma / stroke	Acute BBB disruption; autonomic dysregulation; rapid dysbiosis	Alters brain-to-plasma drug ratio and neuroinflammatory tone	Brain injury-induced dysbiosis may worsen neurological recovery	L3	[8, 9]
	Chronic stress / HPA-axis activation	Elevated cortisol; vagal dysfunction; tight-junction disruption; kynurenine	Can functionally abolish antidepressant behavioral response	Demonstrates strong brain→gut→brain feedback loop; animal evidence only	L3	[10]

Category	Specific Factor / Perturbation	Dominant MGBA Mechanism(s)	Impact on CNS Drug PK/PD	Clinical / Translational Significance	Evidence Level	Source
		activation				
	Aging	Reduced microbial diversity; chronic low-grade inflammation; impaired barrier integrity	Increased toxicity risk and altered CNS exposure	Major contributor to PK variability in elderly polypharmacy	L2	[11]
	Obesity / metabolic syndrome	SCFA dysregulation; endotoxemia; altered bile acid signaling	Synergizes with antipsychotic-induced metabolic toxicity	Amplifies olanzapine-associated weight gain and insulin resistance	L2-L3	[12, 13]
Lifestyle and Nutritional Modifiers	Mediterranean / high-fiber diet	Increased SCFA production; improved barrier integrity; enhanced microbial resilience	Stabilizes absorption and reduces neuroinflammatory signaling	Supports psychotropic responsiveness and metabolic stability	L2-L3	[14, 15]
	Western / high-sugar diet	Reduced SCFA production; mucin degradation; endotoxemia	Promotes dysbiosis and BBB dysfunction	Synergizes with olanzapine-induced obesogenic transition; major dietary confounder (see main text Sec. 7.4)	L2-L3	[1, 12]
	High dietary protein intake	Competition for LAT1 transporters by large neutral amino acids	Reduces intestinal and BBB transport of Levodopa	Important clinically relevant food-drug interaction; actionable *via* dietary counselling	L2	[2, 3]
	Sleep deprivation / circadian disruption	Dysbiosis; increased permeability; altered HPA tone	Intensifies neuroinflammatory drug side effects	Likely contributor to antidepressant non-response	L3	[10, 14]
	Bariatric surgery	Altered anatomy, pH, bile acid circulation, microbiota composition	Alters oral absorption kinetics and exposure variability	Requires reassessment of psychotropic dosing	L2	[7]
Pharmacological Modifiers	Broad-spectrum antibiotics	Microbiota depletion; reduced microbial metabolism; altered glucuronidation	Can increase systemic exposure by removing microbial “metabolic sinks”	Olanzapine exposure increases ~1.8-fold after depletion; (single rodent study; not yet confirmed in humans)	L3	[16]
	Proton pump inhibitors	Increased gastric pH; Enterococcus overgrowth	Intensifies luminal degradation of Levodopa	Important confounder in Parkinson’s disease	L2	[17]
	NSAIDs	Barrier disruption and mucosal inflammation	May increase permeability-dependent PK variability	Potentially amplifies GI toxicity of psychotropics	L4-L5	[6]

Category	Specific Factor / Perturbation	Dominant MGBA Mechanism(s)	Impact on CNS Drug PK/PD	Clinical / Translational Significance	Evidence Level	Source
	Metformin	Strong microbiome remodeling; altered bile acid and SCFA signaling	May indirectly alter psychotropic metabolic response	Important confounder in metabolic syndrome populations	L2	[5]
	Anticholinergic drugs	Reduced GI motility; prolonged luminal residence time	Enhances microbial metabolism and sequestration	Relevant for TCAs and polypharmacy in elderly patients	L3-L4	[9]
	Direct antimicrobial effects of CNS drugs	Broad suppression of commensal taxa	Creates reciprocal feedback loops affecting later efficacy	SSRIs and TCAs suppress ~73% of tested strains (*in vitro*)	L4	[9]
Therapeutic MGBA Modifiers	Psychobiotics / probiotics	Restoration of alpha diversity; modulation of tryptophan and GABA signaling	Potentiate antidepressant efficacy and improve GI symptoms	Emerging adjunctive therapy in depression and PD; several small human trials	L1-L2	[1, 14]
	Prebiotics (B-GOS, inulin)	Promotion of SCFA-producing taxa; barrier reinforcement	Attenuates antipsychotic metabolic toxicity	B-GOS reduces olanzapine-associated weight gain; (rodent data only)	L3	[12, 18]
	Postbiotics / SCFAs	Direct HDAC inhibition; BBB reinforcement; anti-inflammatory signaling	Improves neuroplasticity and target sensitivity	Butyrate restores BBB integrity in experimental systems	L3	[14, 15]
	Fecal microbiota transplantation (FMT)	Ecosystem replacement; removal of high-metabolizer taxa	Alters systemic exposure and CNS response	FMT from good responders increases Levodopa AUC ~2.5-fold; (animal model only; no human FMT trial for this indication yet)	L3	[19]
	Precision microbial enzyme inhibitors	Selective inhibition of bacterial metabolism	Improves systemic bioavailability without affecting host enzymes	AFMT increases Levodopa C_{max} ~1.5-fold; (*in vitro*/animal proof of concept)	L4	[20]
Physiological / Mechanistic Modifiers	Intestinal permeability (“leaky gut”)	Tight-junction disruption; endotoxin translocation	Alters passive absorption and systemic inflammation	Links dysbiosis to neuroimmune activation	L3-L4	[7, 21]
	BBB permeability changes	SCFA depletion; inflammatory cytokines; TMAO signaling	Alters CNS penetration independently of plasma exposure	Important but difficult-to-quantify determinant of CNS drug response	L3	[8, 14]
	Kynurenine pathway	Diversion of tryptophan away from	Creates neurotoxic PD context	Associated with SSRI resistance	L2-L3	[21]

Category	Specific Factor / Perturbation	Dominant MGBA Mechanism(s)	Impact on CNS Drug PK/PD	Clinical / Translational Significance	Evidence Level	Source
	activation	serotonin synthesis	despite therapeutic exposure			
	SCFA depletion	Reduced butyrate and propionate signaling	Weakens BBB integrity and neuroplasticity	Central mechanism in dysbiosis-associated CNS dysfunction	L3	[14, 15]
	Vagal signaling dysfunction	Impaired gut-brain neural communication	Alters emotional processing and drug responsiveness	Important non-PK mechanism of MGBA signaling; animal causal evidence (vagotomy)	L3	[7]
Demographic and Genetic Modifiers	Sex and hormonal state	Hormone-dependent microbial composition and host metabolism	Alters systemic exposure and side-effect profiles	Female PD patients show ~2.1-fold higher Levodopa C_{max}	L2	[17]
	Host CYP450 polymorphisms	Interaction between host metabolism and microbial metabolites	Produces unpredictable systemic exposure	Important foundation for pharmacomicrobiogenomics	L4-L5	[22, 23]
	Polypharmacy	Multiple overlapping microbiome-drug interactions	Creates highly unpredictable PK/PD behavior	Major real-world challenge in psychiatry and neurology	L2	[22]
	Combined gut-liver-brain perturbations	Integrated dysregulation of microbiota, liver metabolism, BBB, and inflammation	Alters exposure-response relationships	Classical host-centric PK models become insufficient	L5	[9]

Table S3. Mechanisms and quantitative data on microbiota-gut-brain axis interactions with selected CNS drugs

Drug	Mechanistic Domain	Pathway / Mechanism	Key Bacteria / Microbial Factors	Metabolites / Molecular Mediators	Animal / In Vitro Data	Human Data	Evidence Level	Clinical / Translational Significance	Source
Levodopa / Carbidopa	Biopharm.	Luminal decarboxylation before absorption	*Enterococcus faecalis*; *Enterococcus faecium*	Tyrosine decarboxylase converts L-DOPA to dopamine	Bacterial TDC exhibits active luminal metabolism of Levodopa	Carbidopa is 14,000–19,000-fold less potent against bacterial tyrosine	L4	Explains persistent peripheral drug loss despite standard carbidopa therapy	[20, 24]

Drug	Mechanistic Domain	Pathway / Mechanism	Key Bacteria / Microbial Factors	Metabolites / Molecular Mediators	Animal / In Vitro Data	Human Data	Evidence Level	Clinical / Translational Significance	Source
						decarboxylase than host AADC			
	Biopharm.	Substrate competition and catalytic preference	*Enterococcus spp.*	TDC preferentially metabolizes tyrosine over Levodopa	Bacterial tyrosine decarboxylase shows 5.0–10.5-fold greater catalytic efficiency for tyrosine	Simultaneous metabolism of tyrosine and Levodopa observed in fecal suspensions	L4	Indicates that high-protein diets may indirectly intensify microbial Levodopa metabolism	[2, 20]
	Biopharm.	Secondary dopamine metabolism	*Eggerthella lenta*	Dopamine dehydroxylase; m-tyramine production	Dopamine dehydroxylase upregulated >2500-fold in dopamine-rich environment	Arg506 SNP predicts ~100% conversion of dopamine to m-tyramine	L4, genotype correlate L2	Microbial metabolism may contribute to hypertensive and autonomic adverse effects	[20]
	Biopharm.	Deamination pathway	*Clostridium sporogenes*	DHPPA [3-(3,4-dihydroxyphenyl)propionic acid]	Active deamination pathway demonstrated in fecal suspensions	Elevated fecal DHPPA in PD patients	L4, genotype correlate L2	DHPPA inhibits ileal motility, creating a self-reinforcing dysmotility loop	[25]
	PK	Precision microbial enzyme inhibition	TDC-expressing *Enterococcus spp.*	alpha-fluoromethyltyrosine (AFMT)	AFMT increases Levodopa C_{max} by ~1.5-fold	N/A	L4	Proof-of-concept for microbiome-targeted adjunctive therapy; not yet in human trials	[20]
	PK	Antibiotic-induced microbiota depletion	Broad microbial depletion	Reduced microbial metabolism	~30% increase in serum AUC; >2-fold increase in striatal dopamine	N/A	L3	Central dopaminergic response exceeds proportional plasma PK change; (animal model	[19]

Drug	Mechanistic Domain	Pathway / Mechanism	Key Bacteria / Microbial Factors	Metabolites / Molecular Mediators	Animal / In Vitro Data	Human Data	Evidence Level	Clinical / Translational Significance	Source
								only)	
	PK	FMT-mediated responder transfer	“Good responder” microbiota	Restoration of low-metabolizer microbial profile	~2.5-fold increase in AUC; 3–4-fold increase in striatal dopamine	N/A	L3	Demonstrates transferable microbiome-dependent responder phenotype; (animal model only)	[19]
	PK	LAT1 transporter competition	Diet-dependent luminal amino acid load	Large neutral amino acids (LNAAs)	N/A	High dietary protein reduces CNS delivery of Levodopa	L2	Competitive transport at gut wall and BBB	[2, 3]
	PK	Delayed gastric emptying and dysbiosis	*Helicobacter pylori*; SIBO-associated taxa	Gastric inflammation; altered pH	N/A	SIBO present in up to 54-67% of PD patients	L2	Delayed ON-time and erratic absorption	[2, 4]
	PD	Clinical dose requirement	High *tdc* abundance microbiota	Excess luminal dopamine production	Wild-type *E. faecalis* lowers plasma levodopa and impairs motor recovery	Fecal *tdc* abundance correlates with daily dose requirement (r = 0.66)	L2, associative (cross-sectional); mechanistic support at L3–L4	Strongest human microbiome-drug-response relationship among CNS drugs	[24]
	PD	Responder paradox	Variable microbial metabolic sink capacity	Variable tyrosine decarboxylase burden	N/A	Tyrosine decarboxylase predicts resistance mainly in moderate	L2	Indicates threshold-dependent microbiome effect	[26]

**Drug**	**Mechanistic Domain**	**Pathway / Mechanism**	**Key Bacteria / Microbial Factors**	**Metabolites / Molecular Mediators**	**Animal / In Vitro Data**	**Human Data**	**Evidence Level**	**Clinical / Translational Significance**	**Source**
						responders			
	PD	Sex-related PK variability	Sex hormone-modulated microbiota	Estrogen-associated PK differences	N/A	Female patients exhibit ~2.1-fold higher Cmax and ~1.8-fold higher AUC	L2	Suggests interaction between hormones, microbiome, and transport/metabolism	[17]
	Clinical	H. pylori eradication	Helicobacter pylori	Gastric pH/inflammation normalization	N/A	AUC increases ~20%; improved motor fluctuations (interventional trial)	L1	Most clinically mature, directly actionable finding for levodopa in this review	[27, 28]
	Clinical	SIBO eradication (rifaximin)	SIBO-associated taxa	Reduced luminal bacterial density	N/A	80% of patients improved motor outcome, reduced OFF time (open-label trial)	L1–L2	Actionable today with standard eradication regimens	[29]
Olanzapine	Biopharm.	Drug-induced structural dysbiosis	*Firmicutes* expansion; *Bacteroidetes* depletion	Altered SCFA production	~1-unit decrease in Shannon diversity; ~75 OTU loss	Significant reduction in alpha diversity in treated patients	L2-L3	Strong evidence for direct drug-on-microbiome effect	[1, 12]
	Biopharm.	Obesogenic transition	*Firmicutes*-dominant profile	Reduced butyrate and propionate	~5-fold increase in *Firmicutes*:*Bacteroidetes* ratio	Similar low-diversity profile observed	L2-L3	Quantitative biomarker associated with metabolic syndrome	[12]

Drug	Mechanistic Domain	Pathway / Mechanism	Key Bacteria / Microbial Factors	Metabolites / Molecular Mediators	Animal / In Vitro Data	Human Data	Evidence Level	Clinical / Translational Significance	Source
						clinically		transition	
	Biopharm.	Commensal depletion	*Blautia*; *Lachnospiraceae*; *Parasutterella*	Reduced SCFA synthesis	~2-fold reduction in *Blautia*/*Lachnospiraceae*; ~60% reduction in *Parasutterella*	N/A	L3	Loss of anti-inflammatory taxa may impair barrier integrity	[12]
	PK	Microbiota depletion and oral bioavailability	Antibiotic-depleted microbiome	Reduced intestinal glucuronidation	~1.8-fold increase in oral bioavailability	N/A	L3 only (no human confirmation)	Establishes microbiota as a major intestinal PK barrier in rats; human translation unconfirmed	[16]
	PK	Intestinal UGT regulation	Microbiota-regulated intestinal enzymes	UGT1A3 modulation	~5-fold reduction in duodenal UGT1A3 mRNA; 2.8-fold hepatic increase	N/A	L3	Gut-wall metabolism more sensitive than hepatic metabolism; (rodent enzyme surrogate)	[16]
	PK	CYP regulation	Microbiota-dependent hepatic signaling	CYP3A1	~1.3-fold increase in hepatic CYP3A1 protein	N/A	L3	Secondary contributor relative to intestinal metabolism	[16]
	PK	SCFA depletion and energy signaling	SCFA-producing taxa depletion	Butyrate; propionate; acetate	>2-fold reduction in butyrate/propionate; 1.78-fold increase in plasma acetate	N/A	L3	Links dysbiosis with adipogenesis and metabolic dysfunction	[12, 18]
	PK	Host metabolic signaling	Adipose and hepatic pathways	GPR43; ACC	Hepatic ACC mRNA increased 2.21-fold; GPR43 decreased ~60%	N/A	L3	Suggests microbiota-mediated lipid storage signaling	[18]
	PD	Weight gain and	Obesogenic	SCFA imbalance;	~30% weight gain in	>7% weight gain	L2-L3	Clinically decisive	[12,

Drug	Mechanistic Domain	Pathway / Mechanism	Key Bacteria / Microbial Factors	Metabolites / Molecular Mediators	Animal / In Vitro Data	Human Data	Evidence Level	Clinical / Translational Significance	Source
		adiposity	microbiota	inflammatory cytokines	rodents	within first year		metabolic toxicity	30]
	PD	Glycemic dysregulation	Dysbiotic inflammatory state	TNF-α; altered acetate signaling	~3-fold increase in glucose	+0.20 mmol/L fasting glucose	L2-L3	Demonstrates diabetogenic transition	[12, 30]
	PD	Lipid dysregulation	Altered microbial-host lipid metabolism	Triglycerides; SCFAs	~2–2.9-fold triglyceride increase	+0.46 mmol/L triglycerides	L2-L3	Strong metabolic syndrome signal	[13, 30]
	PD	Intestinal inflammation	Jejunal inflammatory activation	TNF-α	~2-fold increase in jejunal TNF-α	N/A	L3	Supports gut inflammatory contribution to metabolic toxicity	[12]
	PD	Central receptor adaptation	CNS serotonergic remodeling	5-HT2AR downregulation	~30% decrease in cortical 5-HT2AR protein	N/A	L3	Suggests indirect microbiome-mediated modulation of CNS sensitivity	[18]
	Mechanistic/ Causal	Causal transmission of phenotype	Germ-free / FMT recipient mice	Whole-community transfer	Germ-free mice protected from weight gain; phenotype transmissible via FMT	N/A	L3 (strongest causal animal evidence in olanzapine literature)	Satisfies interventional causality criteria (Sec. 2.5); not yet tested in humans	[31]
Sertraline (SSRIs)	Biopharm.	Microbial depletion and sequestration	Multiple commensal taxa	Strain-dependent drug depletion pathways	20–80% sertraline depletion within 48 h	Inter-individual metabolizer phenotypes in fecal suspensions	L4	Demonstrates variable microbial “metabolic sink”; clinical PK relevance unconfirmed	[32]
	Biopharm.	Intrinsic antimicrobial	Broad commensal microbiota	Membrane-disruptive antimicrobial effects	Suppresses ~73% (185/254) of tested	Reduced alpha diversity during	L4, human corroboration	Establishes reciprocal drug-on-microbiome	[6]

Drug	Mechanistic Domain	Pathway / Mechanism	Key Bacteria / Microbial Factors	Metabolites / Molecular Mediators	Animal / In Vitro Data	Human Data	Evidence Level	Clinical / Translational Significance	Source
		activity			strains	chronic treatment	L2	interaction	
	PK	Stress-induced permeability changes	"Depressive microbiota"	LPS; cytokines	Increased intestinal permeability in stress models	N/A	L3	May alter passive absorption kinetics and inflammatory burden	[33]
	PK	Recovery-associated microbiome normalization	Increased alpha diversity taxa	Restored SCFA signaling	N/A	~1.12-fold increase in Shannon index; ~1.31-fold increase in inverse Simpson index	L2, associative — direction of causality not established (Sec. 2.5)	Suggests microbial recovery accompanies antidepressant response; could be cause or consequence	[34]
	PD	SSRI resistance via inflammatory microbiota	Loss of *Faecalibacterium* and butyrate-producers	IL-6; TNF-α; kynurenine metabolites	Depressive microbiota causes near-100% attenuation of SSRI behavioral efficacy	Elevated IL-6/TNF-α associated with refractory depression	L3 for SSRI class / L5 for sertraline specifically	Strong evidence for PD-context modulation independent of plasma exposure; sertraline-specific replication needed	[1, 33]
	PD	Tryptophan-kynurenine diversion	Dysbiotic inflammatory taxa	Kynurenine; quinolinic acid	Behavioral response restored by 5-HTP supplementation	N/A	L3	Indicates precursor depletion and neurotoxic metabolite accumulation	[21]
	PD	Microglial modulation	CNS immune signaling pathways	TNF-α reduction	Sertraline lowers TNF-α production in microglia models	N/A	L3-L4	Suggests anti-inflammatory CNS effects beyond serotonin reuptake	[6]

Drug	Mechanistic Domain	Pathway / Mechanism	Key Bacteria / Microbial Factors	Metabolites / Molecular Mediators	Animal / In Vitro Data	Human Data	Evidence Level	Clinical / Translational Significance	Source
								inhibition	
	PD	Enteric serotonergic adverse effects	Enteric serotonin signaling	5-HT-mediated motility changes	N/A	Higher diarrhea incidence and strongest SSRI association with microscopic colitis	L2	Clinically relevant gut serotonergic pharmacology	[6]
	Clinical	Adjunctive psychobiotics	Lactobacillus plantarum 299v, PS128; Bifidobacterium longum	Reduced kynurenine; improved neuroimmune signaling	N/A	Reduced kynurenine and improved cognition (299v); modest depression/anxiety improvement (PS128, B. longum)	L1–L2	Most immediately feasible clinical intervention; low risk, modest effect sizes	[35-39]
Amitriptyline (TCAs)	Biopharm.	Broad antimicrobial activity	Multiple commensal strains	Membrane and metabolic disruption	Suppresses ~73% of tested strains	Reduced richness with chronic use	L4, human corraboration L2	Indicates strong reciprocal microbiome remodeling	[9]
	Biopharm.	Structural microbiota shifts	Increased *Bacteroides*; *Alistipes*; *Porphyromonadaceae*	Altered fermentation products	Reduced *Firmicutes*:*Bacteroidetes* ratio after 6 weeks	N/A	L3	Suggests persistent ecological restructuring; (rodent model)	[40]
	PK	Anticholinergic	ENS cholinergic	Prolonged luminal residence	Slower GI transit	N/A	L3-L4	Enhances time	[9]

Drug	Mechanistic Domain	Pathway / Mechanism	Key Bacteria / Microbial Factors	Metabolites / Molecular Mediators	Animal / In Vitro Data	Human Data	Evidence Level	Clinical / Translational Significance	Source
		transit slowing	inhibition	time	demonstrated experimentally			available for microbial metabolism and sequestration; loop not directly quantified	
	PK	Resistome expansion	Gut microbiota resistome	aph3iiiA; mdtK; acrA	Significant increase in resistance genes	N/A	L3	Indicates antimicrobial selection pressure from chronic TCA exposure; human relevance unconfirmed	[40]
	PK	Metabolic pathway modulation	Microbial carbohydrate-metabolism pathways	Membrane transport pathways	Metagenomic pathway alterations detected	CYP2D6 polymorphisms interact with microbial metabolites	L2-L3	May contribute to unpredictable systemic exposure	[9, 41]
	PD	Behavioral and exploratory modulation	*Alistipes*; *Parabacteroides*	Gut-brain signaling metabolites	Altered abundance correlates with exploration behavior	N/A	L3	Suggests microbiota-dependent behavioral modulation; (rodent model)	[40]
	PD	Clinical efficacy in DGBI	Visceral hypersensitivity pathways	ENS modulation; central pain modulation	N/A	IBS-SSS improved by 23.3 points at 3 months (95% CI 4.6-42.0) and 27.0 points at 6 months (95% CI	L1	Clinically meaningful gut-brain therapeutic effect; direct microbiome mechanism not isolated	[41]

Drug	Mechanistic Domain	Pathway / Mechanism	Key Bacteria / Microbial Factors	Metabolites / Molecular Mediators	Animal / In Vitro Data	Human Data	Evidence Level	Clinical / Translational Significance	Source
						7.1-46.9) *vs.* placebo			
	PD	Motility-feedback loop	Anticholinergic dysmotility	Reduced enteric cholinergic signaling	N/A	Chronic constipation may amplify microbial drug transformation	L5	Self-reinforcing gut-drug-microbiome loop; proposed, not directly measured	[9, 40]

Table S4. Interdisciplinary Management of CNS Drug Therapy: Clinical Perturbations, Therapeutic Modifiers, Mechanistic Drivers, and Cross-Specialty Action Points

Drug / Clinical Context	Major Clinical Perturbations	Dominant MGBA Mechanisms	Therapeutic Modifiers / Interventions	Quantitative or Mechanistic Findings	Evidence Level	Cross-Specialty Action Points	Source
Levodopa / Carbidopa in Parkinson's disease	SIBO present in ~54–67% of PD patients; delayed gastric emptying; constipation often precedes motor symptoms by years; H. pylori infection; high-protein diets; PPI-associated Enterococcus overgrowth	Luminal microbial decarboxylation by *Enterococcus faecalis* tyrosine decarboxylase ; dopamine dehydroxylation by *Eggerthella lenta*; LAT1 competition by dietary amino acids; dysmotility prolonging microbial exposure	*H. pylori* eradication; rifaximin for SIBO; protein redistribution diet; precision microbial TDC inhibitors (AFMT); responder-guided FMT; aggressive constipation management; avoidance of dopamine-blocking prokinetics	AFMT increases C*max* ~1.5-fold (L4); antibiotic depletion increases AUC ~30% and striatal dopamine >2-fold (L3); FMT from good responders increases AUC ~2.5-fold and striatal dopamine 3–4-fold (L3); fecal *tdc* abundance correlates with dose requirement (r = 0.66) (L2)	L1 (eradication/SIBO interventions) down to L4 (AFMT)	Neurologists should evaluate GI dysmotility before dose escalation; gastroenterologists should recognize constipation treatment as a PK intervention; pharmacists should monitor PPI and protein-related interactions	[2, 17, 19, 20, 24]

Drug / Clinical Context	Major Clinical Perturbations	Dominant MGBA Mechanisms	Therapeutic Modifiers / Interventions	Quantitative or Mechanistic Findings	Evidence Level	Cross-Specialty Action Points	Source
Levodopa responder stratification	Moderate responders vs good responders	Variable microbial "metabolic sink" capacity; threshold-dependent tyrosine decarboxylase burden	Baseline microbiome profiling; *tdc*-guided stratification; individualized dose escalation	Tyrosine decarboxylase predicts resistance mainly in moderate responders but minimally affects good responders	L2	Pharmacometric stratification should replace empiric dose escalation alone	[26]
Olanzapine-associated metabolic syndrome	Western/high-fat diet; obesity predisposition; low baseline diversity; chronic inflammation; sedentary lifestyle; antibiotic exposure	Drug-induced dysbiosis; 5-fold *Firmicutes*:*Bacteroidetes* increase; SCFA depletion; intestinal inflammation; microbiota-dependent metabolic signaling	Mediterranean/high-fiber diet; B-GOS prebiotic supplementation; metabolic monitoring; microbiome-preserving antibiotic stewardship; exercise interventions	Antibiotic depletion increases bioavailability 1.8-fold (L3, rodent only); >2-fold butyrate reduction (L3); 30% weight gain (L3); 3-fold glucose increase (L3); 2–2.9-fold triglyceride increase (L2–L3, human phenomenology)	L2–L3 (no human microbiome-mechanism confirmation)	Psychiatrists should frame metabolic toxicity as partially microbiome-mediated; gastroenterologists/endocrinologists should participate early in obesity-risk management	[12, 13, 16, 18, 30]
Olanzapine after antibiotic therapy	Broad-spectrum antibiotic exposure; disrupted microbial glucuronidation	Reduced intestinal UGT1A3-mediated first-pass metabolism	Dose reassessment after antibiotic exposure; therapeutic drug monitoring	~1.8-fold increase in systemic bioavailability after microbiota depletion (single rodent study)	L3	Clinical pharmacologists should anticipate transient exposure surges after microbiota disruption	[16]
Olanzapine and prebiotic co-therapy	SCFA depletion and adipose signaling dysregulation	Reduced butyrate/propionate; altered acetate-GPR43 signaling	B-GOS and SCFA-restoring interventions	B-GOS attenuates olanzapine-induced weight gain without altering central 5-HT2AR blockade (rodent model)	L3	Supports adjunctive microbiome-targeted metabolic protection strategy	[18]

Drug / Clinical Context	Major Clinical Perturbations	Dominant MGBA Mechanisms	Therapeutic Modifiers / Interventions	Quantitative or Mechanistic Findings	Evidence Level	Cross-Specialty Action Points	Source
Sertraline in major depressive disorder	Pro-inflammatory "depressive microbiota"; chronic stress; elevated IL-6/TNF-α; IBS/IBD overlap; sleep deprivation	Kynurenine pathway activation; microglial priming; low butyrate; increased permeability; reciprocal antimicrobial drug effects	Psychobiotics; anti-inflammatory dietary interventions; stress reduction; tryptophan/5-HTP normalization; microbiome restoration	Depressive microbiota can nearly completely block SSRI-class (fluoxetine-demonstrated) behavioral response (L3); recovery associated with ~1.12–1.31-fold diversity increase (L2, associative)	L2-L3	Psychiatrists should evaluate inflammatory/dysbiotic background in treatment-resistant depression; gastroenterologists should recognize SSRI effects on enteric serotonergic signaling	[1, 33, 34]
Sertraline-associated GI toxicity	Microscopic colitis risk; diarrhea; IBS overlap; chronic enteric serotonergic stimulation	Enteric SERT inhibition; microbiome depletion; altered motility	Slow dose titration; psychobiotic support; differentiation between transient enteric adaptation and true inflammatory pathology	Sertraline suppresses ~73% of tested gut strains (L4); strongest SSRI association with microscopic colitis (L2)	L2	Gastroenterologists should distinguish drug-induced diarrhea from IBD exacerbation	[6]
Amitriptyline in disorders of gut-brain interaction (DGBI)	IBS; chronic visceral hypersensitivity; constipation-predominant states; polypharmacy	Anticholinergic slowing of transit; prolonged luminal exposure; altered microbiota composition	Low-dose initiation; fiber supplementation; bowel regimen optimization; avoidance of additive anticholinergic burden	IBS-SSS improved by 23.3 points at 3 months (95% CI 4.6-42.0) and 27.0 points at 6 months (95% CI 7.1-46.9) *vs*. placebo (randomized controlled trial)	L1	Gastroenterologists and psychiatrists should coordinate dose titration and monitor anticholinergic toxicity; already actionable in clinical practice	[9]
Amitriptyline-associated microbiome remodeling	Chronic TCA exposure	Reduced *Firmicutes*:*Bacteroidetes* ratio; resistome expansion; altered	Monitoring during prolonged therapy; microbiome-supportive diet	Increased aph3iiiA, mdtK, and acrA resistance genes (rodent model)	L3	Long-term TCA use may contribute to antimicrobial resistance ecology; human relevance unconfirmed	[40]

Drug / Clinical Context	Major Clinical Perturbations	Dominant MGBA Mechanisms	Therapeutic Modifiers / Interventions	Quantitative or Mechanistic Findings	Evidence Level	Cross-Specialty Action Points	Source
		Bacteroides/*Alistipes* abundance					
CNS drugs in inflammatory bowel disease (IBD)	Chronic mucosal inflammation; increased permeability; cytokine activation	Gut-derived IL-6/TNF-α signaling; BBB permeability changes; altered target sensitivity	Biologics (anti-TNF); psychobiotics; careful psychotropic selection	IBD associated with 30–40% prevalence of depression/anxiety (association, not proven causal direction)	L2	Psychiatrists should consider inflammatory burden when evaluating antidepressant resistance	[6, 7]
Neurotrauma / stroke context	Acute BBB disruption; rapid dysbiosis within hours; systemic inflammation	Brain→gut autonomic signaling; permeability increase; neuroimmune activation	Early enteral nutrition; probiotic support; inflammation control	Brain injury-associated dysbiosis worsens neuroinflammation and recovery (animal model)	L3	Neurocritical care should incorporate gut-barrier preservation strategies as an emerging, not yet standard, consideration	[8, 9]
Chronic stress / HPA-axis activation	Elevated cortisol; chronic anxiety/depression; sleep disruption	Tight-junction disruption; dysbiosis; vagal dysfunction; kynurenine activation	Stress reduction; psychobiotics; sleep normalization; dietary fiber enrichment	Chronic stress can functionally abolish SSRI response in preclinical models	L3	Psychiatric treatment resistance should include assessment of stress-mediated MGBA dysfunction as a research-informed hypothesis	[10]
Polypharmacy and aging	PPIs, NSAIDs, metformin, antibiotics, anticholinergics; inflammaging	Altered pH; Enterococcus overgrowth; permeability changes; cumulative dysbiosis	Medication reconciliation; deprescribing; microbiome-aware pharmacotherapy	PPIs promote *Enterococcus* expansion and intensify levodopa degradation	L2	Elderly patients are highly vulnerable to microbiome-mediated PK variability	[17]
Bariatric surgery and CNS drug exposure	Altered anatomy, pH, transit time, bile acid signaling	Gut hormone remodeling; altered microbiota; modified absorption kinetics	Post-surgical dose reassessment; therapeutic drug monitoring	Major PK variability expected after RYGB and sleeve gastrectomy	L2	Psychiatry and bariatric teams should coordinate psychotropic dose adjustment	[17]
Barrier-restoration strategies	Leaky gut; reduced SCFA production; BBB dysfunction	Tight-junction disruption; endotoxemia; microglial activation	SCFAs; prebiotics; psychobiotics; FMT; Mediterranean diet	Butyrate restores BBB tight-junction integrity in experimental systems	L3	Barrier restoration may improve both PK stability and PD responsiveness	[14, 15, 42]

Drug / Clinical Context	Major Clinical Perturbations	Dominant MGBA Mechanisms	Therapeutic Modifiers / Interventions	Quantitative or Mechanistic Findings	Evidence Level	Cross-Specialty Action Points	Source
General pharmacometric implications	Inter-individual variability in microbiome composition and function	Dynamic microbiome-drug-host interactions	Integration into PBPK/PD models; longitudinal biomarker monitoring	Classical host-centric PK models fail to capture microbiome-mediated exposure variability	L5 (framework, not yet implemented)	Precision neuropharmacology requires microbiome-aware dosing frameworks; presently a research agenda (see main text Sec. 7.2, 7.5)	[9, 22]

References:

1. Abidin ZZ, Hein ZM, Nassir CMNCM, Shari N, Ramli MDC. Pharmacological modulation of the gut-brain axis: psychobiotics in focus for depression therapy. Frontiers in Pharmacology. 2025;16:1665419, doi: 10.3389/fphar.2025.1665419.
2. Menozzi E, Schapira AHV. The Gut Microbiota in Parkinson Disease: Interactions with Drugs and Potential for Therapeutic Applications. Cns Drugs. 2024;38(5):315-31, doi: 10.1007/s40263-024-01073-4.
3. Zhong Z, Ye M, Yan F. A review of studies on gut microbiota and levodopa metabolism. Front Neurol. 2023;14:1046910, doi: 10.3389/fneur.2023.1046910.
4. Dzidic-Krivic A, Kusturica J, Sher EK, Selak N, Osmancevic N, Karahmet Farhat E, et al. Effects of intestinal flora on pharmacokinetics and pharmacodynamics of drugs. Drug Metab Rev. 2023;55(1-2):126-39, doi: 10.1080/03602532.2023.2186313.
5. Long-Smith C, O'Riordan KJ, Clarke G, Stanton C, Dinan TG, Cryan JF. Microbiota-Gut-Brain Axis: New Therapeutic Opportunities. Annu Rev Pharmacol Toxicol. 2020;60:477-502, doi: 10.1146/annurev-pharmtox-010919-023628.
6. Hatamnejad MR, Baradaran Ghavami S, Shirvani M, Asghari Ahmadabad M, Shahrokh S, Farmani M, et al. Selective serotonin reuptake inhibitors and inflammatory bowel disease; Beneficial or malpractice. Front Immunol. 2022;13:980189, doi: 10.3389/fimmu.2022.980189.
7. Macpherson AJ, Pachnis V, Prinz M. Boundaries and integration between microbiota, the nervous system, and immunity. Immunity. 2023;56(8):1712-26, doi: 10.1016/j.immuni.2023.07.011.
8. Hasan A, Scuderi SA, Capra AP, Giosa D, Bonomo A, Ardizzone A, et al. An Updated and Comprehensive Review Exploring the Gut-Brain Axis in Neurodegenerative Disorders and Neurotraumas: Implications for Therapeutic Strategies. Brain Sci. 2025;15(6):654, doi: 10.3390/brainsci15060654.
9. Xu T, Roy SP, Hadia R, Annu, Bhowmik S, Panchal A. The gut-brain axis in pharmacology: microbiome-driven modulation of CNS drugs and neuropsychiatric outcomes. Front Pharmacol. 2026;17:1826681, doi: 10.3389/fphar.2026.1826681.
10. Rusch JA, Layden BT, Dugas LR. Signalling cognition: the gut microbiota and hypothalamic-pituitary-adrenal axis. Frontiers in Endocrinology. 2023;14:1130689, doi: 10.3389/fendo.2023.1130689.
11. Zhang F, Ding K, Zhang LM, Liu DY, Dong X, Wang MN, et al. The role of the gut microbiota in neuropsychiatric disorders and therapy. Ageing Res Rev. 2025;112:102894, doi: 10.1016/j.arr.2025.102894.
12. Kamath S, Hunter A, Collins K, Wignall A, Joyce P. The atypical antipsychotics lurasidone and olanzapine exert contrasting effects on the gut microbiome and metabolic function of rats. British Journal of Pharmacology. 2024;181(22):4531-45, doi: 10.1111/bph.16507.
13. Qian L, He X, Liu Y, Gao F, Lu W, Fan Y, et al. Longitudinal Gut Microbiota Dysbiosis Underlies Olanzapine-Induced Weight Gain. Microbiol Spectr. 2023;11(4):e0005823, doi: 10.1128/spectrum.00058-23.
14. Loh JS, Mak WQ, Tan LKS, Ng CX, Chan HH, Yeow SH, et al. Microbiota-gut-brain axis and its therapeutic applications in neurodegenerative diseases. Signal Transduction and Targeted Therapy. 2024;9(1):37, doi: 10.1038/s41392-024-01743-1.
15. Motataianu A, Serban G, Andone S. The Role of Short-Chain Fatty Acids in Microbiota-Gut-Brain Cross-Talk with a Focus on Amyotrophic Lateral Sclerosis: A Systematic Review. International Journal of Molecular Sciences. 2023;24(20):15094, doi: 10.3390/ijms242015094.
16. Cussotto S, Walsh J, Golubeva AV, Zhdanov AV, Strain CR, Fouhy F, et al. The gut microbiome influences the bioavailability of olanzapine in rats. EBioMedicine. 2021;66:103307, doi: 10.1016/j.ebiom.2021.103307.
17. Miyaue N, Yamamoto H, Liu S, Ito Y, Yamanishi Y, Ando R, et al. Association of Enterococcus faecalis and tyrosine

decarboxylase gene levels with levodopa pharmacokinetics in Parkinson's disease. NPJ Parkinsons Dis. 2025;11(1):49, doi: 10.1038/s41531-025-00903-6.

18. Kao AC, Spitzer S, Anthony DC, Lennox B, Burnet PWJ. Prebiotic attenuation of olanzapine-induced weight gain in rats: analysis of central and peripheral biomarkers and gut microbiota. Transl Psychiatry. 2018;8(1):66, doi: 10.1038/s41398-018-0116-8.
19. Ai P, Xu S, Yuan Y, Xu Z, He X, Mo C, et al. Targeted Gut Microbiota Modulation Enhances Levodopa Bioavailability and Motor Recovery in MPTP Parkinson's Disease Models. Int J Mol Sci. 2025;26(11):5282, doi: 10.3390/ijms26115282.
20. Rekdal VM, Bess EN, Bisanz JE, Turnbaugh PJ, Balskus EP. Discovery and inhibition of an interspecies gut bacterial pathway for Levodopa metabolism. Science. 2019;364(6445):eaau6323, doi: 10.1126/science.aau6323.
21. Kearns R. Gut-Brain Axis and Neuroinflammation: The Role of Gut Permeability and the Kynurenine Pathway in Neurological Disorders. Cellular and Molecular Neurobiology. 2024;44(1):64, doi: 10.1007/s10571-024-01496-z.
22. Kumar N, Das J, Vanangamudi M, Mojgani N, Islam A, Sridhar SB, et al. Modulating drug response through the gut microbiome: pathways to precision therapy. Archives of Microbiology. 2026;208(4):163, doi: 10.1007/s00203-025-04688-3.
23. Liu LX, Wang HY, Minichino A, Xiao R, Du YM, Wang L, et al. Mapping the reciprocal interactions between antidepressants and the gut microbiome: novel targets for the personalisation and optimization of drug response. Molecular Psychiatry. 2026;31(4):1992-2004, doi: 10.1038/s41380-025-03345-x.
24. van Kessel SP, Frye AK, El-Gendy AO, Castejon M, Keshavarzian A, van Dijk G, et al. Gut bacterial tyrosine decarboxylases restrict levels of levodopa in the treatment of Parkinson's disease. Nat Commun. 2019;10(1):310, doi: 10.1038/s41467-019-08294-y.
25. van Kessel SP, de Jong HR, Winkel SL, van Leeuwen SS, Nelemans SA, Permentier H, et al. Gut bacterial deamination of residual levodopa medication for Parkinson's disease. Bmc Biology. 2020;18(1):137, doi: 10.1186/s12915-020-00876-3.
26. Feng Y, Zhang R, Wen G, Xie L, Chen T, Liu W. The role of gut microbiota tyrosine decarboxylases in levodopa pharmacokinetics: insights from a levodopa challenge test. BMC Neurol. 2025;25(1):460, doi: 10.1186/s12883-025-04428-6.
27. Pierantozzi M, Pietroiusti A, Galante A, Sancesario G, Lunardi G, Fedele E, et al. Helicobacter pylori-induced reduction of acute levodopa absorption in Parkinson's disease patients. Ann Neurol. 2001;50(5):686-7, doi: 10.1002/ana.1267.
28. Pierantozzi M, Pietroiusti A, Brusa L, Galati S, Stefani A, Lunardi G, et al. Helicobacter pylori eradication and l-dopa absorption in patients with PD and motor fluctuations. Neurology. 2006;66(12):1824-9, doi: 10.1212/01.wnl.0000221672.01272.ba.
29. Fasano A, Bove F, Gabrielli M, Petracca M, Zocco MA, Ragazzoni E, et al. The role of small intestinal bacterial overgrowth in Parkinson's disease. Mov Disord. 2013;28(9):1241-9, doi: 10.1002/mds.25522.
30. Pillinger T, McCutcheon RA, Vano L, Mizuno Y, Arumuham A, Hindley G, et al. Comparative effects of 18 antipsychotics on metabolic function in patients with schizophrenia, predictors of metabolic dysregulation, and association with psychopathology: a systematic review and network meta-analysis. Lancet Psychiatry. 2020;7(1):64-77, doi: 10.1016/S2215-0366(19)30416-X.
31. Morgan AP, Crowley JJ, Nonneman RJ, Quackenbush CR, Miller CN, Ryan AK, et al. The antipsychotic olanzapine interacts with the gut microbiome to cause weight gain in mouse. PLoS One. 2014;9(12):e115225, doi: 10.1371/journal.pone.0115225.
32. Zimmermann M, Zimmermann-Kogadeeva M, Wegmann R, Goodman AL. Mapping human microbiome drug metabolism by gut bacteria and their genes. Nature. 2019;570(7762):462-7, doi: 10.1038/s41586-019-1291-3.

33. Ramsteijn AS, Jasarevic E, Houwing DJ, Bale TL, Olivier JD. Antidepressant treatment with fluoxetine during pregnancy and lactation modulates the gut microbiome and metabolome in a rat model relevant to depression. Gut Microbes. 2020;11(4):735-53, doi: 10.1080/19490976.2019.1705728.
34. Liskiewicz P, Pelka-Wysiecka J, Kaczmarczyk M, Loniewski I, Wronski M, Baba-Kubis A, et al. Fecal Microbiota Analysis in Patients Going through a Depressive Episode during Treatment in a Psychiatric Hospital Setting. Journal of Clinical Medicine. 2019;8(2):164, doi: 10.3390/jcm8020164.
35. Chen HM, Kuo PH, Hsu CY, Chiu YH, Liu YW, Lu ML, et al. Psychophysiological Effects of Lactobacillus plantarum PS128 in Patients with Major Depressive Disorder: A Preliminary 8-Week Open Trial. Nutrients. 2021;13(11), doi: 10.3390/nu13113731.
36. Ho YT, Tsai YC, Kuo TBJ, Yang CCH. Effects of Lactobacillus plantarum PS128 on Depressive Symptoms and Sleep Quality in Self-Reported Insomniacs: A Randomized, Double-Blind, Placebo-Controlled Pilot Trial. Nutrients. 2021;13(8), doi: 10.3390/nu13082820.
37. Kazemi A, Noorbala AA, Azam K, Eskandari MH, Djafarian K. Effect of probiotic and prebiotic vs placebo on psychological outcomes in patients with major depressive disorder: A randomized clinical trial. Clin Nutr. 2019;38(2):522-8, doi: 10.1016/j.clnu.2018.04.010.
38. Pinto-Sanchez MI, Hall GB, Ghajar K, Nardelli A, Bolino C, Lau JT, et al. Probiotic Bifidobacterium longum NCC3001 Reduces Depression Scores and Alters Brain Activity: A Pilot Study in Patients With Irritable Bowel Syndrome. Gastroenterology. 2017;153(2):448-59 e8, doi: 10.1053/j.gastro.2017.05.003.
39. Rudzki L, Ostrowska L, Pawlak D, Malus A, Pawlak K, Waszkiewicz N, et al. Probiotic Lactobacillus Plantarum 299v decreases kynurenine concentration and improves cognitive functions in patients with major depression: A double-blind, randomized, placebo controlled study. Psychoneuroendocrinology. 2019;100:213-22, doi: 10.1016/j.psyneuen.2018.10.010.
40. Zhang W, Qu W, Wang H, Yan H. Antidepressants fluoxetine and amitriptyline induce alterations in intestinal microbiota and gut microbiome function in rats exposed to chronic unpredictable mild stress. Transl Psychiatry. 2021;18(11):131, doi: 10.1038/s41398-021-01254-5.
41. Camilleri M, Sarnelli G, Andresen V, Greenwood-Van Meerveld B, Howden CW, Izzo AA, et al. Pharmacological, Pharmacokinetic, and Pharmacogenomic Aspects of Disorders of Gut-Brain Interaction. Gastroenterology. 2026;170(6):1152-70, doi: 10.1053/j.gastro.2026.02.010.
42. O'Riordan KJ, Moloney GM, Keane L, Clarke G, Cryan JF. The gut microbiota-immune-brain axis: Therapeutic implications. Cell Reports Medicine. 2025;6(3):101982, doi: 10.1016/j.xcrm.2025.101982.
